# Phytochemistry, Bioactivities and Traditional Uses of *Michelia × alba*

**DOI:** 10.3390/molecules27113450

**Published:** 2022-05-26

**Authors:** Kian-Kai Cheng, Muhammad Helmi Nadri, Nor Zalina Othman, Siti Nor Azlina Abd Rashid, Ying-Chin Lim, Hong-Yeng Leong

**Affiliations:** 1 Innovation Centre in Agritechnology, Universiti Teknologi Malaysia, Muar 84600, Malaysia; chengkiankai@utm.my (K.-K.C.); muhammad.helmi@utm.my (M.H.N.); norzalina@utm.my (N.Z.O.); sitinorazlina@utm.my (S.N.A.A.R.); 2 Faculty of Engineering, School of Chemical and Energy Engineering, Universiti Teknologi Malaysia, Skudai 81300, Malaysia; 3 Faculty of Applied Sciences, Universiti Teknologi MARA, Shah Alam 40450, Malaysia; limyi613@uitm.edu.my

**Keywords:** *Magnolia alba*, therapeutic, bioactive compounds

## Abstract

*Michelia × alba* (*M. alba*) is a flowering tree best known for its essential oil, which has long been used as a fragrance ingredient for perfume and cosmetics. In addition, the plant has been used in traditional medicine in Asia and dates back hundreds of years. To date, there is a limited number of publications on the bioactivities of *M. alba*, which focused on its tyrosinase inhibition, antimicrobial, antidiabetic, anti-inflammatory, and antioxidant activities. Nevertheless, *M. alba* may have additional unexplored bioactivities associated with its bioactive compounds such as linalool (72.8% in flower oil and 80.1% in leaf oil), α-terpineol (6.04% flower oil), phenylethyl alcohol (2.58% flower oil), β-pinene (2.39% flower oil), and geraniol (1.23% flower oil). Notably, these compounds have previously been reported to exhibit therapeutic activities such as anti-cancer, anti-inflammation, anti-depression, anti-ulcer, anti-hypertriglyceridemia, and anti-hypertensive activities. In this review paper, we examine and discuss the scientific evidence on the phytochemistry, bioactivities, and traditional uses of *M. alba*. Here, we report a total of 168 *M. alba* biological compounds and highlight the therapeutic potential of its key bioactive compounds. This review may provide insights into the therapeutic potential of *M. alba* and its biologically active components for the prevention and treatment of diseases and management of human health and wellness.

## 1. Introduction

*Michelia × alba* (*M. alba*), also known as *Magnolia × alba* (D.C.) Figlar, is a perennial plant commonly found in tropical regions including Thailand, Indonesia, Malaysia, and China. The plant is well known for its flower essential oil, which is commonly produced through steam distillation [1]. Its scent is often described as sugary, floral, champagne like with a slight herbal scent. *M. alba* essential oil is registered under the category of GRAS “generally recognized as safe” (FEMA Number: 3950, CAS: 92457-18-6) under Section 201(s) of the Federal Food, Drug, and Cosmetic Act by Flavor and Extract Manufacturers Association (FEMA). The essential oil is a common fragrance ingredient found in skin care, perfume, and cosmetics. In addition, it is also widely used as a flavoring agent in baked goods, beverages, condiments, frozen dairy, gelatins/pudding, meat products, and soft candy.

To date, there are about 200 patents reported on *M. alba* essential oil. In addition, there is an increasing trend in the number of *M. alba* research publications since 2001 (https://link.lens.org/3k1hpsF0pmc, accessed on 12 January 2022). There are accumulating studies reporting its potential bioactivities including tyrosinase inhibition, photoprotection, anti-stress, anti-diabetic, antioxidant, anti-gout, and antimicrobial activities. Some of the studies focused on bioactive ingredients found in *M. alba* such as linalool (72.8% in flower oil and 80.1% in leaf oil), α-terpineol (6.04% in flower oil), phenylethyl alcohol, β-pinene (2.39% in flower oil), and geraniol (1.23% of flower oil) [2]. Notably, linalool is the primary component found in *M. alba* which is also found in lavender and jasmine oils [2] Numerous studies on linalool have reported its anti-cancer, anti-inflammatory, neuroprotective activity, anti-hypertensive activity, anti-ulcer, anti-hypertriglyceridemia, anti-psoriasis, antidepressant, and anti-diarrheal activities. In 2018, a new compound named Michelaine (C_16_H_9_NO_4_) was discovered from the flower of *M. alba* [3]. However, the bioactivities of Michelaine that are uniquely found in *M. alba* are not established. Taken together, the pharmacological potential of *M. alba* has not been fully uncovered. Therefore, the current review aimed to compile the important findings of *M. alba* and its bioactive compounds and further highlights the potential of *M. alba* as a drug discovery candidate for the future.

## 2. Botanical Description

### 2.1. Taxonomic Classification and Nomenclature

*M. alba* is a hybrid of *Magnolia champaca* (L.) Baill. ex Pierre and *Magnolia montana* (Blume) Figlar (International Plant Names Index (IPNI) Life Sciences Identifier (LSID): urn:lsid:ipni.org:names:20011680-1) [4]. The taxonomic classification and nomenclature of *Michelia × alba* are as follows:
Kingdom : PlantaeDivision : MagnoliophytaClass : MagnoliopsidaOrder : MagnolialesFamily : MagnoliaceaeGenus : *Michelia*Species : *Michelia × alba*

### 2.2. Botanical Name

#### 2.2.1. Synonyms

*Michelia × alba*, *Michelia alba* D.C., Figlar, *Magnolia* (D.C.) *Figlar* × *alba*, *Magnolia champaca* × *Magnolia montana*, *Magnolia longifolia* Blume, Verh. Bat. Gen., *Magnolia longifolia* var. racemosa Blume, Fl. Java Magnol., *Magnolia champaca* auct. non Linne, *Sampacca × longifolia* (Blume) Kuntze.

#### 2.2.2. Common Name

Bailan (白兰), White Sandalwood, White Champaca, White Chempaka, Cempaka Putih, Chempaka Puteh, Cempaka Gading, Chempak, Chempaka, Pecari Putih, White Jade Orchid Tree, tjempaka bodas, tjampaka momero, tjempaka mawuro, tjampaka pote, tjempaka putih, Djeumpa gadeng, petjari putih, sampaka kulo, s. mopoesi, bunga edga kebo, patene, bunga edja mapute, tjapaka bobudo, tjapaka bobulo.

### 2.3. Distribution and Plant Morphology

*M. alba* belongs to the genus *Michelia* (Magnoliaceae) which consists of about 30 species [5]. *M. alba* is commonly cultivated in tropical and sub-tropical regions such as Southeast Asia, and it is widely cultivated in China, especially in southern regions such as Fujian, Guangdong, Hainan, Guangxi, and Yunnan [5,6,7]. The flowering plant is also native to Thailand, Indonesia, and Malaysia. In these countries, *M. alba* is widely cultivated as an ornamental plant [1].

**Figure 1:**
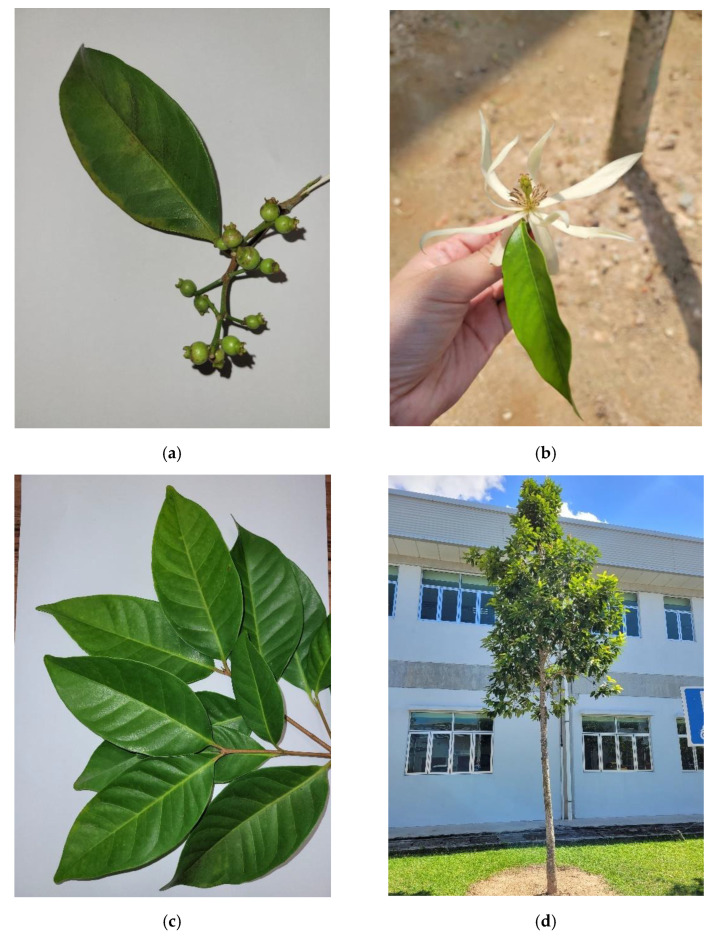
Photos of *M. alba*. (**a**) carpels, (**b**) flower, (**c**) leaves, and (**d**) *M. alba* plant (photo taken on *M. alba* planted in Universiti Teknologi Malaysia Pagoh Campus).

*M. alba* is an annual flowering plant that can grow as high as 20 m in high humidity regions [5]. The morphological features and pictures of the *M. alba* plant (Figure 1) are shown in Table 1. The plant starts to produce flowers at a height ranging from 10 to 15 m and the flowering time usually begins in the evening (8–9 pm). The flower scent is said to spread quickly and widely which will be faded in the afternoon. Therefore, the harvesting activities for *M. alba* flowers are usually conducted in the evening and at dawn [1,5].

## 3. Phytochemistry

*M. alba* is best known for its essential oil. Essential oils are first obtained by steam distillation in the Middle Ages by Arabs with characteristics of intense aroma. Its volatile compounds are formed by secondary metabolites extracted from aromatic plants [10]. Essential oil can be extracted from the flowers, leaves, or stems of *M. alba* [11]. Solvent extraction, steam distillation, and enfleurage are some of the common extraction methods for *M. alba* essential oil. Steam distillation is commonly used in commercial *M. alba* essential oil extraction because the processing method is convenient and economical [1,12].

One of the earliest studies in 1982 reported 24 constituents in *M. alba* flower essential oil namely, β-bisabolene, δ-cadinene, Δ~3-carene, camphene, cis-caryophyllene, α-cubebene, β-cubebene, ο-cymene, trans-carveol, eugenol, isoaristolene, limonene, linalool, cis-linalool oxide, trans-linalool oxide, methyl, methyl 2-methylbutyrate, methyl isoeugenol, β-myrcene, ocimene, α-phellandrene, β-pinene, β-selinene, and α-ylangene [13]. The findings, together with two other comprehensive studies by [14,15] on the chemical constituents of essential oil extracted from *M. alba*’s flower (F), leaves (L), and stem (S) have identified a total of 168 constituents in which 102 constituents were from the flower (F), 101 from leaves (L), and 77 from the stem (S) (Supplementary Data S1).

**Figure 2:**
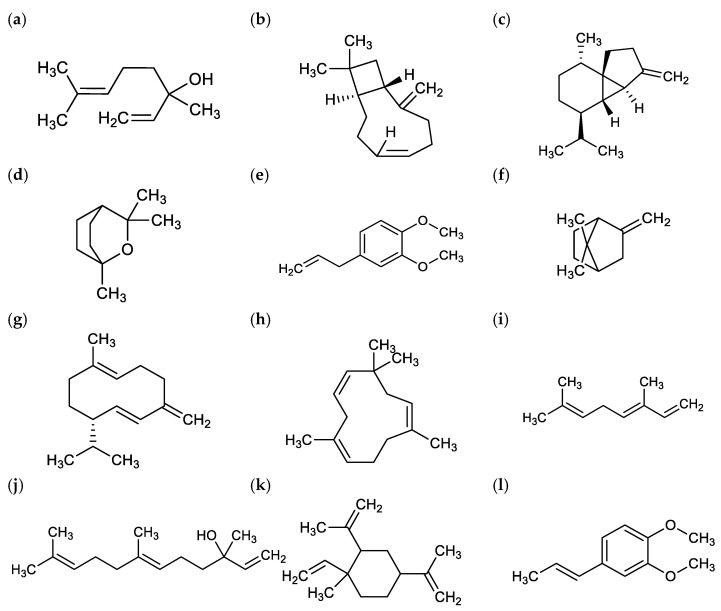
The main aroma constituents in the essential oil of flower, stem, and leaves of *M. alba.* (**a**) Linalool, (**b**) caryophyllene, (**c**) β-cubebene, (**d**) eucalyptol, (**e**) eugenol methyl ether, (**f**) α-fenchene, (**g**) germacrene D, (**h**) α-humulene, (**i**) (E)-ocimene, (**j**) nerolidol, (**k**) 2,4-diisopropenyl-1-methyl-1-vinylcyclohexane, (**l**) isoeugenyl methyl ether [11,14,15,16].

Terpenoids are secondary metabolites essential for plant growth and development which contribute to the aroma, flavor, and color of the plant. They protect the plant from environmental stress, pests, and microorganism [16]. Most of the chemical constituents identified are from monoterpene and sesquiterpene. There are differences in the chemical constituents of extracted *M. alba* oils in published reports. However, the main constituents are similar, i.e., linalool, caryophyllene, β-cubebene, eucalyptol, eugenol methyl ether, α-fenchene, germacrene D, α-humulene, (E)-ocimene, nerolidol, 2,4-diisopropenyl-1-methyl-1-vinylcyclohexane, and isoeugenyl methyl ether (Figure 2) [11,14,15,16]. *M. alba* contains relatively high levels of linalool in various plant parts. It was reported that the linalool concentration in leaves, flowers, and young twigs was about 0.21 to 0.65%, 1.63 to 4.89%, and 0.43%, respectively [7]. Linalool is especially high in juvenile *M. alba* flowers, which was reported to be ten-fold higher than in leaves and stems. In addition, the fallen leaves of *M. alba* also have high concentrations of linalool [7].

In addition to extensive studies on the chemical constituents of the *M. alba* essential oil, the molecular structures of the constituents isolated from different plant parts were also studied with the aim to understand the chemotaxonomy and identification of the medicinal properties of non-volatile compounds. The constituents from *M. alba* flower, leaves, and stem were extracted with methanol and analyzed with chromatography while compound structures were characterized and identified with spectral analyses. A total of 42 constituents were identified from the extracts of different plant parts of *M. alba* (Supplementary Data S2). Out of the total 42 constituents, 19 constituents were isolated from the flower (F), 21 isolated from leaves (L), and 20 from the stem (S) [3,17,18]. Different chemical constituents have been identified from various parts of *M. alba* extracts. Some of the major compounds discovered include aporphines, amide, benzenoids, and steroids. In 2018, a study by [3] discovered a new compound named Michelaine (C_16_H_9_NO_4_) in the flower of *M. alba*.

## 4. Bioactivities *M. alba* Extracts

To date, there are a number of reported bioactivities of *M. alba* extracts, including tyrosinase inhibition and photoprotective activities, antimicrobial, antidiabetic, anti-inflammatory, and antioxidant activities.

### 4.1. Tyrosinase Inhibition and Photoprotective Activities

Tyrosinase is an enzyme that catalyzes the production of melanin. Overexpression of tyrosinase can cause various dermatologic disorders including post-inflammatory hyperpigmentation [19]. This condition is not only aesthetically undesirable, but it may affect patients’ emotions and quality of life [20]. It has been reported that (−)-N-formylanonaine, a purified compound isolated from *M. alba* inhibits in vitro mushroom tyrosinase activity in a dose-dependent manner with an IC_50_ value of 74.3 µM [21]. This inhibition activity is comparable to an established tyrosinase inhibitor, kojic acid with a recorded IC_50_ value of 69.4 µM. In addition, a molecular docking study suggests the tyrosinase inhibitory effect of (−)-N-formylanonaine may be due to its ability to chelate two copper ions in the active site of tyrosinase [21]. In an epidermal melanocytes cell culture study, (−)-N-formylanonaine was found to inhibit human tyrosinase activity at concentration ranges of 10–200 µM. Consequently, melanin content was also found reduced in cells treated with this compound at the same concentrations with an EC_50_ value of 90 µM [21]. On the other hand, a cell culture study showed the potential of α-terpineol as a skin whitening agent. Treatment of α-terpineol (at 100 and 200 µM) was reported to reduce melanin content and tyrosinase activity in B16 cells stimulated with α-melanocyte-stimulating hormone (α-MSH) [22]. Importantly, α-terpineol at concentrations of 100 and 200 µM did not affect B16 cell viability. In the same cell model, α-terpineol also prevented oxidative stress by reducing cellular malondialdehyde and increased cellular GSH levels. Tyrosinase inhibition activity of phenylethyl alcohol has been reported by [23] using in vitro mushroom tyrosinase assay. This compound was isolated from *Rosa rugosa* Thunb. var. plena Regal tea. Phenylethyl alcohol inhibits mushroom tyrosinase activity in a dose-dependent manner with an IC_50_ value of 315 ± 13 μg/mL. However, kojic acid (positive control) showed more potent inhibitory activity with an IC_50_ value of 80 ± 17 μg/mL.

Exposure to solar ultraviolet (UV) radiation on the skin leads to photoaging. This condition is characterized by the degradation of extracellular matrix (ECM) proteins which include type 1 collagen, elastin, proteoglycans, and fibronectin. This will then damage the connective tissue and reduce the elasticity of the dermis [24]. Irradiation of UV promotes the formation of reactive oxygen species, induces the expression of the mitogen-activated (MAP) kinase signaling pathway, and upregulates the expression of matrix metalloproteinase (MMP)-1, MMP-3, and MMP-9 [24]. *M. alba* extract inhibits the expression of the three matrix metalloproteinases in UVB-induced activation of p-JNK and p-ERK on cultured human fibroblasts cells and consequently restores total collagen synthesis [24].

### 4.2. Antimicrobial Activity

Natural products from microorganisms, plants, animals, and algae may serve as a good source of novel antimicrobial compounds [25]. A number of phytochemical extracts from flowers (including *M. alba*) or their essential oils have been reported to have potential antimicrobial activities for treating various diseases [26,27,28].

#### 4.2.1. Antibacterial and Anti-Fungal Activities

The antimicrobial activity of the Magnolia family may be due to the presence of various bioactive constituents extracted from different parts of the plants. *M. alba* is rich in carbohydrates, alkaloids, terpenoids, flavonoids, tannins, steroids, and phenols. It has been used not only in traditional medicine but also as a potential antiseptic for the prevention and treatment of microbial infections [29]. *M. champaca* seed and flower extracts were reported to inhibit the microbial growth of *Aeromonas hydrophila*, *E. coli*, *Edwardsiella tarda*, *Flavobacterium spp*., *Klebsiella pneumonia*, *Salmonella typhi*, *Vibrio alginolyticus*, *V. parahaemolyticus*, *V. cholerae*, *Pseudomonas aeruginosa*, *Staphylococcus aureus*, *Bacillus subtilis*, and *Shigella dysenteriae* [28,30,31].

*M. alba* and *M. champaca* exhibited comparable effects on antibacterial inhibition of *S. aureus*, *E. coli*, and *Psedumonas aeuroginosa* (Table 1). Notably, the antimicrobial activity of leaf oil was found stronger than that of stem oil on growth inhibition of *S. aureus* ATCC 13709; *E. coli* ATCC 25922; *Candida albican* ATCC 10231 [32]. In addition, [33] reported the *M. alba* dichloromethane leaf extract with 76.6% linalool gave a better inhibitory effect on the growth of *Psedumonas aeuroginosa*, *C. albican*, and *Fusarium oxysporium* compared with the n-pentane flower extract (PF) with 63.2% linalool. The dichloromethane leaf extract was an efficient *C. albicans* growth inhibitor, while *F. oxysporium* was more susceptible to the dichloromethane flower extract [33].

The methanol extract of *M. alba* bark was reported to inhibit the growth of *C. Verruculosa*, which causes leaf spot disease on rice [34]. It was found that the antifungal activity of *M. alba* essential oil was strongly correlated with linalool and caryophyllene which are known to inhibit the growth of *Aspergillus flavus* [11]. In addition, the antifungal activity of *M. alba* oil against the growth of *Aspergillus niger*, *Aspergillus flavus*, *Penicillium sp.*, *Rhizopus sp.*, *Fusarium sp.*, and *Cladosporium sp.* was demonstrated through the application of the oil to the surface of bamboo paper packaging boxes [35].

#### 4.2.2. Antiparasitics

Anti-parasitic agents have various applications including organic or conventional livestock production systems. Domestic animals such as cattle, pigs, dogs, and cats carry harmful parasites such as *Trypanosoma cruzi* [36]. *T. cruzi* can easily infest livestock animals and becomes an endemic that causes a devastating impact on the livestock industry worldwide. The trypanocidal constituents from the ethanol extract of the bark of *M. alba* (Table 2) showed good antiparasitic activity against *T. cruzi* [37]. In addition, the pharmacological activities of −anonaine from *M. alba* have been reviewed by Li and colleagues [38] which showed that the compound gives a significant inhibitory effect against *Plasmodium falciparum* that causes malaria in humans. The compound also protected red blood cells against *P. falciparum*. As the compound shows low cytotoxicity in the Chinese Ovarian cell line, it may be a potential phytochemical compound for the treatment of malaria (The Pharmacological Ac.).

### 4.3. Anti-Diabetic Activity

The anti-diabetic potential of *M. alba* essential oil was demonstrated through the inhibition of α-amylase, a digestive enzyme found in saliva and pancreatic juice. This enzyme digests complex carbohydrates into oligosaccharides and disaccharides. α-amylase inhibitors delay the hydrolysis of carbohydrates in the intestines [41]. Therefore, inhibition of α-amylase may serve as a therapeutic target for the prevention and medical treatment of diabetes [42]. The essential oil from *M. alba* inhibits α-amylase activity with an IC_50_ value of 0.67 mg/mL. The inhibition activity is lower than the positive control, acarbose, which showed an IC_50_ value of 0.06 mg/mL. GC-MS analysis of essential oil indicated the presence of β-linalool (65.03%) as its major compound [43]. A molecular docking study suggests the β-linalool forms hydrogen bonds with His-299 and Asp-300 residues of α-amylase with a binding energy of − 5.20 kcal/mol [43]. On the other hand, aldose reductase is an enzyme that converts glucose into sorbitol in the presence of nicotinamide adenine dinucleotide phosphate (NADPH). Accumulation of sorbitol in the cells has been associated with the development of diabetic neuropathy. Aldose reductase inhibitor can be used as a target to reduce the concentration of sorbitol in the cells. Lee et al. [44] reported that *M. alba* flower extract dose-dependently inhibits aldose reductase activity with an IC_50_ value of 1.98 µg/mL.

### 4.4. Anti-Inflammatory Activity

Gout is an inflammatory arthritis characterized by the accumulation of uric acid in the blood and further deposited within visceral tissues and joints. Xanthine oxidase catalyzes the oxidation of hypoxanthine to xanthine and its further conversion to uric acid. A number of plant extracts and their metabolites showed inhibition against xanthine oxidase [45]. Leaves extract of *M. alba* inhibits in vitro xanthine oxidase activity by 22.49% at a concentration of 100 µg/mL. The observed inhibition activity is higher than *Gliricidia sepium* which showed 6.94% at the same concentration. However, the inhibition activity of *M. alba* extract was found lower than several medicinal plants such as *Antegonon leptopus* (59%), *Mimosa pudica* (62.36%), and *Vitex negundo* (38.4%) at 100 µg/mL [46].

### 4.5. Antioxidant Activity

Oxidative stress has been recognized as one of the classical risk factors for human diseases such as cardiovascular diseases, cancers, and neurodegenerative diseases [47]. In biological systems, macromolecules such as lipids, proteins, and nucleic acids are prone to oxidation upon exposure to free radicals. Excessive production of free radicals and a low antioxidant level collectively contribute to oxidative stress leading to a negative impact on physiological function.

In 2018, Zheng and colleagues reported antioxidant activity and phenolics profile of 65 edible flowers in China [48]). In the study, the *M. alba* flower was extracted using a mixture of acetone/water/acetic acid (70:29.5:0.5, *v*/*v*/*v*). Its 2,2-diphenyl-1-picrylhydrazyl (DPPH) results showed that the extract recorded 58.22 µmol Trolox equivalents (TE)/g sample) on a dry weight basis, higher than several other edible flowers including *Panax pseudoginseng* (15.18 µmol TE/g sample), *Prunella vulgaris* (21.39 µmol TE/g sample), and *Siraitia grosvenorii* (21.03 µmol TE/g sample) [48]. In the 2,2′-azinobis-(3-ethylbenzothiazoline-6-sulfonate) (ABTS) and ferric reducing antioxidant power (FRAP) assays, the extract showed 111.54 µmol TE/g of dry weight sample and 15.51 mmol of Fe2+E/100 g sample, respectively [48]. In another study, petroleum ether extract of *M. alba* flower showed DPPH free radical scavenging activity with an IC_50_ value of 0.7155 mg/mL. This inhibition activity is higher than in several other aromatic plants such as *Plumeria alba* and *Cananga odorata* [49].

## 5. Therapeutic Potential of *M. alba* Metabolites

Despite the limited number of reported bioactivities of *M. alba*, its bioactive compounds have been associated with numerous therapeutic effects, suggesting the additional unexplored bioactivities of *M. alba*. In this section, we discuss the bioactivities of the major compounds (including linalool, α-terpineol, and β-pinene, (−)-anonaine, geraniol, (−)-N-formylanonaine, and geraniol) found in *M. alba* which may provide insight into the additional therapeutic potential of *M. alba*.

### 5.1. Anti-Cancer Activity

The potential anti-cancer activities of *M. alba* may be attributed to two of its bioactive compounds, including linalool and (−)-anonaine. Linalool is the most abundant component in the flower oil (72.8%) and leaf oil (80.1%) of *M. alba* [2]. It was reported to exhibit anticancer properties and induce apoptosis in the U937 leukemia cells and HeLa cervical cancer cells [50]. The anticancer activity may be associated with the expression of cyclin-dependent kinase inhibitors (CDKIs) as well as the non-expression of cyclin-dependent kinases (CDK) activity [50]. The results are consistent with a study by Gu and colleagues which showed that linalool is an activator of the p53/CDKI axis and inhibited the growth of a number of leukemia cells with wild-type p53 (including Kasumi-1, HL-60, Molt-4, and Raji cells) [51].

In another in vitro study, linalool treatment led to the selective inhibition of the growth of RPMI 7931 human melanoma cells, but not the NCTC 2544 normal keratinocytes cell lines [52]. In the study, the anti-cancer activity was attributed to the enhanced caspase 3 activity in the linalool-treated melanoma cells. Furthermore, linalool also showed anti-cancer properties on HCT 116 human colon cancer cell line and inhibited the growth of a human xenografted tumor mouse model with no observable side effects [53]. In the study, its anti-proliferative effect was linked to the induction of hydroxyl radical generation [53]. Using an in vitro cell culture assay, Cherng and colleagues also demonstrated anti-proliferative activity of linalool towards bladder (J82), lung (H661 and H520), cervix (HeLa), stomach (AGS), skin (BCC-1/KMC), bone (U2OS), mouth (OSCC-1/KMC), and kidney (RTCC-1/KMC) carcinoma cell lines [54]. Furthermore, linalool treatment was also found able to suppress the growth of breast (T-47D), colorectal (SW620), liver (Hep G2), lung (A549), and prostate (DU145, 22Rv1) cancer cell lines [55,56,57]. In addition to *M. alba*, linalool is also found present in several other plants. A coriander seed oil extract that contained 56.5% linalool was reported to decrease the viability of the HepG2 human liver cancer cell line, together with an increase in reactive oxygen species (ROS) and perturbation in mitochondrial activities [58]. In addition, treatment with linalool and *lavender angustifolia* essential oil (30.5% linalool) significantly inhibited the proliferation of DU145 and PC-3 human prostate cancer cells and suppressed tumor growth in a xenograft model with PC-3 cell transplantation [59].

On the other hand, (−)-anonaine is a bioactive compound found in the leaves of *M. alba* with reported anti-cancer activity [38]. Previously, Chen and colleagues showed that (−)-anonaine treatment significantly inhibited cell growth and resulted in anti-migratory and DNA-damaging effects on the H1299 human lung carcinoma cells [60]. Furthermore, (−)-anonaine was found to induce DNA damage and reduce viability in HeLa cells but not in noncancerous Madin-Darby canine kidney (MDCK) and Vero cells [61]. The anti-cancer property of (−)-anonaine on HeLa cells may be linked with induced nitric oxide and ROS, loss of mitochondrial membrane potential, activation of caspase 3, 7, 8, and 9, as well as increased expression of apoptosis-related proteins (including Bax, Bcl-2, and p53), and increased poly (ADP-ribose) polymerase cleavage [61].

Taken together, these findings highlight the anti-cancer properties of linalool and (−)-anonaine, the key bioactive components of *M. alba*, which may have the potential to be developed into therapeutic bioproducts. Notably, Han and colleagues developed linalool-incorporated nanoparticles which demonstrated anti-cancer properties in cell lines (including HeyA8, A2780, and SKOV3ip1) and patient-derived xenograft mouse models of epithelial ovarian cancer [62].

### 5.2. Anti-Inflammatory Activity

Previously, the anti-inflammatory potential of (−)-linalool, a major component in *M. alba* oil, has been demonstrated in a rat model of inflammation. In the study, (−)-linalool significantly inhibited carrageenin-induced edema in rats [63]. The anti-inflammatory effect was found more profound than (±) linalool [63]. In a further study, Peana and colleagues showed that (−)-linalool inhibited the production and release of nitric oxide (NO) in lipopolysaccharide-stimulated J774.A1 macrophages [64]. In addition, treatment with lavender essential oil which contained 32.52% linalool was found effective in reducing inflammatory response induced by carrageenan and by croton oil in a rat model [65].

Psoriasis is a common skin problem and is considered a chronic inflammatory disease. The hallmark feature of psoriasis includes hyperproliferation and abnormal differentiation of keratinocytes [66]. Psoriasis leads to negative physical and emotional impacts on the patients. Recently, Rai and colleagues demonstrated the anti-psoriasis potential of linalool. Using a cell-based assay, linalool at concentrations of 0.01 and 0.1% significantly reduced pro-inflammatory cytokines including TNF-α and IL-6 in LPS-activated murine macrophage cells [67]. In addition, an in silico study also showed that linalool may have good binding against several psoriasis targets such as IL-17, IL-22, IL-23, IL-17a, IL-17b, IL-17f, IL-22α1, and IL-22α2.

Furthermore, the anti-psoriasis activity of linalool was investigated in vivo using Imiquimod (IMQ) induced psoriasis-like skin inflammation in a BALB/c mouse model. Administration of linalool at concentrations of 1 and 2% improved IMQ-induced psoriasis mice with improved body weight and decreased ear thickness. Psoriasis area and severity index (PASI) is a gold standard for quantitative assessment of the severity and extent of psoriasis. PASI scoring is calculated based on parameters including thickness, scaling, and erythema of the skin. The recovery percentage of >50 in PASI scores can be considered a significant improvement in psoriasis [68]. Topical application of linalool at 1 and 2% showed 54.76 and 64.28% recovery, respectively, in PASI scores [67]. Linalool at a concentration of 2% also showed significant recovery in Th-1 cytokines (TNF-α and IL-1β) and Th-17 cytokines (IL-17 and IL-22). In the study, immunohistochemistry analysis also showed that linalool reduced the expression of NF-κB, IL-17, and CCR6 in the skin tissue sections. These results suggest the potential of linalool against psoriasis.

On the other hand, geraniol, an *M. alba* bioactive compound, was found able to improve osteoarthritis conditions in in vitro and in vivo assays. Treatment of geraniol (40 and 80 µM) attenuated the expression of proinflammatory markers (including iNOS, COX-2, PGE2, NO release, TNF-α, and IL-6) expression in IL-1β-activated mouse chondrocytes [69]. This inhibitory activity was found pronounced at gene and protein levels. Extracellular matrices compose of two essential components which are type II collagen and aggrecan. Exposure of IL-1β suppresses the expression of these components. Treatment of geraniol at concentrations of 40 and 80 µM significantly reduced the deleterious effect of IL-1β in mouse chondrocytes. A disintegrin and metalloproteinase with thrombospondin motifs 5 (ADAMTS-5) and matrix metalloproteinases-9 (MMP-9) are degradative enzymes that exhibit proteolytic activity on ECM, hence promoting osteoarthritis development. Exposure to IL-1β enhanced the expression of both enzymes. However, treatment of geraniol reduces the degradation of ECM induced by IL-1β. The mechanism of action by which geraniol exhibits such effect is known to involve inhibition of PI3K/Akt/NF-κB and MAPK activation. The study indicated the potential role of geraniol in inhibiting inflammatory markers that may be useful for the therapy of osteoarthritis.

### 5.3. Therapeutic Potential of *M. alba* on Mental Health Disorders

A few recent publications suggested the potential therapeutic effects of bioactive compounds in *M. alba* on mental health disorders such as depression, cognitive impairment, convulsion, Alzheimer’s disease, and stress. The bioactive compounds involved include linalool, α-terpineol, and β-pinene.

Depression is a common mental disorder that severely affects the quality of life. It has been reported that more than 264 million people suffer from this illness [70]. It is characterized by an inability to experience pleasure, fatigue, lack of appetite, loss of weight, insomnia, and loss of ability to think or concentrate. Previously, anti-depressant activity of linalool was demonstrated on a rat model using the splash test and forced swimming test. The splash test was used to identify depressive symptoms, as characterized by an increase in time for rats to initiate self-cleaning and decreased total self-cleaning time. Intraperitoneal (IP) injection of linalool at 30 mg/kg significantly decreased the latency of self-cleaning initiation and increased the total time of self-cleaning [71]. Linalool treatment showed a similar pattern to those rats that receive fluoxetine, an anti-depressant drug. In the forced swimming test, rats receiving an IP injection of linalool at a dose of 30 mg/kg showed significantly shorter immobility time as compared to the control [71]. These findings indicated the anti-depressant potential of linalool.

Recently, the neuroprotective activity of linalool had been demonstrated using a hemiparkinsonian rat model [72]. In the study, 6-hydroxydopamine (6-OHDA) was used to induce models of Parkinson’s disease (PD). Treatment of linalool at doses of 25, 50, and 100 mg/kg significantly improved the behavioral alteration in the 6-OHDA lesion group as compared to the untreated 6-OHDA group, as measured by apomorphine-induced rotations, open field, and force swimming tests. In addition, linalool treatment also attenuated the level of dopamine, 3,4-dihydroxyphenylacetic acid and homovanillic acid (HVA) compared to the untreated 6-OHDA lesion group. These data suggested the potential neuroprotection activity of linalool.

Cognitive impairment is one of the characteristics of a pathological condition of vascular dementia, Alzheimer’s disease, and ischemic stroke. In a transient cerebral ischemia rat model, α-terpineol has been reported to provide protection against impairment of hippocampal synaptic plasticity and spatial memory [73]. Treatment with α-terpineol at 100 mg/kg through IP injection significantly reduced the escape latency during training trials as evaluated by the Morris water maze method. A similar dose of α-terpineol also facilitated the induction of long-term potentiation in the hippocampus which was persistent over two hours as determined by electrophysiological recording. In addition, α-terpineol (at 100 and 200 mg/kg) also significantly reduced lipid peroxidation in the hippocampus of the rat model (*p* < 0.05) [73]. These results indicated the potential of this compound in improving cerebral ischemia cognitive impairment.

In another study, anti-convulsant activity of α- and β-pinene have been evaluated using male Swiss albino rats. To determine the lethal dose of both compounds, α- and β-pinene at a concentration of 2000 mg/kg were administered through IP injection to the rats and observed for a duration of 10, 30, 60, 120, 180, and 240 min, and for 14 consecutive days [74]. From the result, both compounds showed a mild sedation effect without any toxicity or death recorded. To determine the anti-convulsant effect, Swiss Albino rats were treated with α-pinene or β-pinene at concentrations of 100–400 mg/kg, with an equimolar combination of α- and β-pinene (400 mg/kg), reference drug diazepam (2 mg/kg), or vehicle solution (0.5% Tween 80 in saline). After 1 h of administration, 80 mg/kg pentylenetetrazol was injected through IP to stimulate seizures in the animal model. Animals were sacrificed at the end of the experiment. The results showed that compounds at a concentration of 400 mg/kg significantly reduced the seizure intensity as compared to the control group (*p* < 0.05). In addition, the equimolar mixture of both compounds (400 mg/kg) significantly enhanced the latency of the first convulsion. Furthermore, β-pinene (400 mg/kg) and mixture (400 mg/kg) also significantly increased the time of mortality of animals as compared to the controls (*p* < 0.05).

Acetylcholinesterase inhibitors have been used to treat Alzheimer’s disease. Recently, the potential acetylcholinesterase and butyrylcholinesterase inhibition activities of β-pinene have been demonstrated using a molecular docking technique. The results showed that β-pinene has a binding affinity of −6.4 kcal/mol towards acetylcholinesterase [75]. In the binding site, β-pinene interacts with hydrophobic amino acids such as Trp-83, Phe-329, Phe-330, and His-439. In butyrylcholinesterase, β-pinene showed a binding affinity of −5.6 kcal/mol with Tyr-329, Phe-326, Trp-79, and Ala-325 interactions. In addition, in silico ADMET (absorption, distribution, metabolism, and excretion toxicity) analysis predicted that β-pinene can be absorbed orally and passed through the blood–brain barrier without considerable side effects [75].

Analysis of electroencephalogram (EEG) showed that there are distinctive brain wave patterns associated with brain states and functions. Alpha waves are known for relaxed and passive attention brain state. In contrast, beta waves are observed during alertness, anxiety dominance, and attention [76,77]. A recent study using human subjects reported that inhalation of *M. alba* essential oil reduces beta waves and increases fast alpha wave activity [77]. The inhalation of linalool, the main component in the essential oil, also showed similar effects [77]. Collectively, *M. alba* essential oil and its key bioactive ingredient, linalool, may be useful as an anti-stress and sedative agent.

### 5.4. Antioxidant Activity

In addition to the reported antioxidant activity of *M. alba* extract, a number of studies had reported antioxidant activities of *M. alba* metabolites such as (−)-N-formylanonaine, α-terpineol, linalool, and geraniol. The (−)-N-formylanonaine was found to inhibit DPPH free radical scavenging with an IC_50_ value of 121.4 µM, as compared with ascorbic acid which showed a higher scavenging activity (IC_50_ value: 52.1 µM) [21]. In a metal chelating ability assay, (−)-N-formylanonaine recorded an IC_50_ value of 262.1 µM. The reducing power of (−)-N-formylanonaine (100 µM) was reported at 0.56, a moderate value compared with 1.28 for 3-tert-butyl-4-hydroxyanisole (BHA), a standard antioxidant [21]. In addition, Wang et al. [21] reported the isolation of several bioactive compounds including michephyll A from the leaves of *M. alba* [78]. Analysis of the antioxidant activity of michephyll A (100 µM) using ABTS free radical scavenging and metal chelating activities showed moderate antioxidant activity with 40.5 and 55.2% inhibitions, respectively [78].

On the other hand, Chaudhari and colleagues reported the antioxidant activity of α-terpineol using ABTS and DPPH free radical scavenging assays [79]. The study showed that α-terpineol has an IC_50_ value of 7.25 µL/mL for ABTS analysis and scavenges DPPH free radical with an IC_50_ value of 57.86 µL/mL. Encapsulation of α-terpineol with chitosan nanoemulsion enhanced DPPH free radical scavenging activity with an IC_50_ value of 39.57 µL/mL [79]. In addition to an in vitro assay, the antioxidant activity of α-terpineol has been reported in a rat model fed with a high-fat diet [80]. The intake of both R-(+)- and (–)-α-terpineol enantiomers at a dose of 50 mg/kg significantly reduced serum and liver thiobarbituric acid reactive substances (TBARS) in the rat model [80].

In a human study, the DPPH analysis of blood plasma showed that linalool inhalation increased antioxidant activity in healthy adults and patients with carpal tunnel syndrome (CTS) [81]. In addition, linalool affects CTS patients by reducing blood pressure and pulse rate which may serve as a useful intervention for CTS. In a cell culture assay, linalool acts as an antioxidant against UVB-induced ROS generation and prevents depletion of antioxidant enzymes [82]. It protects cultured skin cells from UVB-induced oxidative stress by modulating MAPK and NF-κB signaling. Furthermore, Jabir and colleagues showed that linalool (50 µg/mL) exhibits high scavenging activity (50.57%) in an in vitro DPPH assay and 56.36% scavenging activity in an H_2_O_2_ scavenging assay [83].

The antioxidant potential of geraniol has been demonstrated using cellular and animal models. In a DPPH assay, geraniol exhibits free radical scavenging activity with an IC_50_ value of 663 nmol [84]. Geraniol (100 nmol) also attenuated the effect of 2,2′-azobis(2-methylpropionamidine) (AAPH)-induced lipid peroxidation in rat brain tissue and sciatic nerve homogenates. In addition, geraniol at a concentration of 10 µM for 24 h reduced reactive oxygen species and hydroperoxides levels, but not GSH in glucose-induced oxidative stress cultured in SH-SY5Y human neuroblastoma cells [84].

Accumulation of aluminum in tissue can induce oxidative stress and toxicity. Exposure to aluminum chloride (AlCl_3_) (70 mg/kg, IP) in male Wistar rats increased malondialdehyde (MDA) activity and reduced total antioxidant and glutathione peroxidase activities [85]. The oxidative stress induced by AlCl_3_ in rats was found reversed by co-treatment of geraniol (100 mg/kg, orally) with AlCl_3_ as indicated by reduced MDA activity and increased total antioxidant and glutathione peroxidase activities. In another in vivo study, oral administration of geraniol (150 mg/kg) for 14 days increased total antioxidant activity, GSH levels, and normalizing MDA level in liver ischemia-reperfusion injury rats through activation of the Nrf2/HO-1 signaling pathway [86].

### 5.5. Potential Anti-Hypertensive, Anti-Diabetic and Anti-Hypertriglyceridemia Activities

Linalool, the primary bioactive compound in *M. alba*, was reported to exhibit potential anti-hypertensive, anti-diabetic, and anti-hypertriglyceridemia activities. Oral administration of linalool at a dose of 100 mg/kg showed anti-hypertensive activity in spontaneously hypertensive rats (SHR) [87]. Linalool at the same dose also improved cardiovascular parameters such as decreasing cardiac hypertrophy, enhancing vasodilation, and decreasing vasoconstriction. Cytokine IL-10 also decreased in the linalool-treated SHR model, indicating anti-inflammatory activity [87]. There are no symptoms of drug toxicity observed in treated rats. Interestingly, linalool complexed with β-cyclodextrin exhibited higher anti-hypertensive activity than a pure form [87]. This improvement may be due to the role of β-cyclodextrin in providing greater bioavailability as compared to uncomplexed linalool [87].

In addition, Jun and colleagues [88] reported that linalool reduced triglycerides levels in a dose-dependent manner. At 1 mM, linalool reduced 37% triglyceride level as compared to the control group in cultured hepatocytes. In gene and protein expression assays, linalool (1 mM) increased PPARα expression by 1.9- to 1.8-fold, respectively. Consequently, linalool also resulted in significant upregulation of lipid metabolism genes including FATP4, ACS1, ACOX 1, UPC2, and LPL expression as well as downregulation of APOC3 expression. Jun and colleagues [88] also performed in vivo experiments to study the effect of linalool on plasma triglycerides concentration. The result showed that oral administration of linalool (100 mg/kg body weight/day) on Western diet-fed C57BL/6J mice and human apo E2 mice for 3 weeks reduced plasma triglycerides concentration by 31 and 50%, respectively. Based on metabolomic analysis, linalool treatment reduced saturated fatty acids in mice [88]. These results indicated the potential of linalool in regulating the hepatic transcriptome and plasma metabolome.

### 5.6. Anti-Ulcer Activity

Peptic ulcer refers to the erosion of mucosa in the stomach (gastric) or small intestine (duodenal). It is well known that the use of NSAIDs and *Helicobacter pylori* infection are among the main causes of ulcers. Previous studies have demonstrated the therapeutic activity of natural products against peptic ulcers [89]. For example, the anti-ulcer activity of linalool was studied using ethanol-induced gastric ulcer in a mouse model. Treatment of 10, 20, and 40 mg/kg of linalool significantly reduced gastric lesion area by 85.50, 76.26, and 89.35%, respectively, compared to vehicle-treated mice [90]. In comparison, positive control mice receiving carbenoxolone at a concentration of 100 mg/kg exhibited reduced lesion area by 93.92%. In acetic acid-induced gastric ulcers, treatment of oral linalool (40 mg/kg) for 14 days also significantly reduced gastric lesion area by 48% without showing any sign of toxicity in mice. The incorporation of linalool with β-cyclodextrins further improved the gastroprotection effect of linalool alone against gastric ulcers [90].

In addition, the anti-ulcer activity of geraniol has been tested using an in vivo assay [91]. In the study, acetic acid injection and *H. pylori* inoculation were used to induce ulcers in adult male Sprague-Dawley rats. After induction of ulcers, the rats were administered with geraniol (at 15 and 30 mg/kg), vehicle, or a mixture of standard drugs (amoxicillin, 50 mg/kg; clarithromycin, 25 mg/kg; and omeprazole, 20 mg/kg) twice daily for a duration of 14 days. After treatments, the animals were sacrificed for evaluation of gastric ulcers. The results showed a significant reduction in ulcer index (UI) when rats were treated with geraniol at concentrations of 15 mg/kg (UI: 2.20) and 30 mg/kg (UI: 2.00) when compared to the control group with *H. pylori* (UI: 4.13). Geraniol also increased GSH levels and reduced MPO activity and gastric secretion in this rodent model. Histopathological evaluation revealed that treatment of geraniol (30 mg/kg) significantly reduced lesion score (score: 3.5) as compared to the *H. pylori* control group (9.0). Through Giemsa staining, geraniol at both concentrations reduced *H. pylori* load in the gastric mucosa as compared to controls with the *H. pylori* group. This study showed the gastro-protective effect of geraniol against gastric ulcers and *H. pylori* in rats.

### 5.7. Anti-Diarrheal Activity

Previously, a study showed that treatment of α-terpineol at all tested concentrations (6.25, 12.5, 25, and 50 mg/kg) significantly reduced castor oil-induced diarrheal in mice (47.11, 65.95, 55.62, and 10.33%, respectively) [92]. The same doses of α-terpineol also reduced stool total weight (55, 48, 44, and 24%, respectively). In addition, α-terpineol also reduced gastrointestinal transit (31%). Mice receiving 12.5 mg/kg α-terpineol also significantly reduced the effect of PGE2-induced diarrheal (39%). Furthermore, α-terpineol reduced fluid formation and loss of chloride ions by interacting with GM1 receptors and cholera toxin, which resulted in increased uptake of intestinal fluids, therefore showing anti-diarrheal activity of this compound in the mouse model [92].

### 5.8. Anti-Asthmatic Activity

Treatment of α-terpineol (0.75 and 1.25 mmol/L) was found to induce relaxation in the tracheal smooth muscle of a guinea pig model [93]. Several derivatives of α-terpineol, including 2-(4-methylcyclohex-3-enyl)propan-2-yl 2-(2-hydroxyethylthio)acetate, showed even higher relaxation activity than α-terpineol. The compound also showed anti-asthmatic activity in the ovalbumin-induced asthmatic rat model by decreasing airway resistance (RL) and enhancing the dynamic compliance of the airway. Together, the anti-asthmatic potential of α-terpineol and its derivatives warrant further study and development.

## 6. Traditional Uses and Potential Application of *Michelia alba*

### 6.1. Ethnomedicinal Uses

*Michelia* species have been used as a cancer treatment by natives in India and China. In India, *M. champaca* has been used to treat abdominal tumors, while in China, *M. hypoleuca* and *M. officinalis* were used for the treatment of carcinomatous sores and leukemia [17,94]. *M. alba* is well known for its wide variety of medicinal and traditional uses from the flower, barks, roots, and leaves. *M. alba* has a long history of safe use in traditional medicine. It has been used for the treatment of fever, syphilis, gonorrhea, malaria, and prevention of bronchitis, prostatitis, and leucorrhea, and the flower was used traditionally as an abortive agent in Asian countries [5,6,16,17]. In addition, the plant is also believed to be useful in treating abnormal vaginal discharge and irregular menstrual cycles [95]. The essential oil of *M. alba* is used to treat inflammatory conditions and cancer [11]. *M. alba* was also found to suppress cough and treat expectorant and bronchitis [1].

The flower of *M. alba* has a strong alluring and sweet floral scent. This property makes it a common garland in traditional ceremonies and religious offerings in Asia. In addition, the *M. alba* flower was often used in aromatherapy for mental disorders treatment and the dried flowers are also used in Thailand for heart and nerve care as well as motion sickness [1,96,97]. Besides medicinal use, *M. alba* leaves extraction was reported to be utilized as a repellent agent [98].

In Thailand, the flowers and leaves of *M. alba* were said to reduce mucus production, prevent bad breath, and break down kidney stones [34]. Furthermore, the bark of *M. alba* was reported to use for the treatment of malaria, syphilis, and gonorrhea, and to reduce fever symptoms and the white fragrant flower is used traditionally as an abortive agent [5]. In the Philippines, *M. alba* is used to treat inflammation disorders including gout, rheumatism, and asthma [46].

### 6.2. Food Additive and Preservative

Bioactive compounds (e.g., linalool) isolated from *M. alba* have been approved as a natural food additive and are deliberated to be generally recognized as safe (GRAS) by the FDA as a synthetic flavoring substance and adjuvant in food for human consumption including as an ingredient in animal drugs, feeds, and related products [99]. In 2017, Songsamoe and colleagues developed antifungal fragrant brown rice using the vapor phase of *M. alba* oil [39]. As bioactive compounds in *M. alba* such as linalool, caryophyllene and β-elements may affect the sensory quality leading to an anti-stress effect [11], the researchers demonstrated that the application of *M. alba* oil in the vapor phase is capable of improving consumer acceptance and preference for cooked brown rice while significantly controlling the growth of *A. flavus*, i.e., molds in brown rice [35]. In addition, the application of *M. alba* oil or isolated compounds such as linalool may help to protect natural packaging materials made from high carbon content materials from the growth of molds [35]. Taken together, *M. alba* is a potential natural food preservative and may enhance food flavor.

## 7. Conclusions

**Figure 3:**
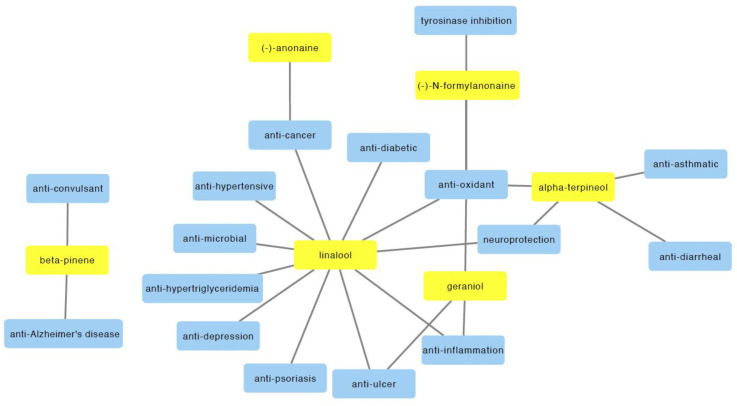
A summary of bioactivities of selected bioactive compounds in *M. alba*.

In summary, this review summarizes the published findings on the phytochemistry, bioactivities, and traditional uses of *M. alba*. A summary of its potential bioactivities is shown in Figure 3. However, some of the reported in vitro studies involved treatments with high concentrations of *M. alba* compounds. It should be noted that future studies are crucial to identify the precise therapeutic ranges of these compounds in humans based on different age groups. Notably, there is accumulating evidence showing the multiple therapeutic potential and applications of linalool, the primary bioactive compound in *M. alba* oil. Nevertheless, the pharmacological potential of *M. alba* has not been fully uncovered, which may be further studied by a combination of in vitro and in vivo bioactivity assays, network pharmacology, and in silico bioactivity prediction methods. In addition, further preclinical and clinical studies are required to validate the therapeutic potential of *M. alba* and its bioactive compounds, their interaction, safety, efficacy, and underlying mechanism of action for future clinical applications.

## Figures and Tables

**Table 1:** Morphological features of *Michelia alba*’s leaves, stems, flowers, fruits, and seeds with photo [6,8,9].

Plant Part	Morphological Features
Tree	Height: 10–30 m Bark: Grey
Leaves	Color: Green Arrangement: coriaceous, glabrous above, sparsely pubescent below, elliptic to obovate-elliptic Size: 15–35 cm × 5.5–11 cm Apex: acuminate Acumen: 0.7–30 mm Shape: simple and elliptical
Twigs/petiole	Petiole color: grayish Petiole length: 15–50 mm Arrangement: sparsely appressed puberulent to glabrous
Flowers	Odor: aromatic, especially after dark Diameter: 5 cm Arrangement: Tepals: 30–55 mm Lanceolate: 3–5.5 × 0.3–0.5 mm Stamens: 8–10 mm long Filaments: 1–1.5 mm long Color: white or cream
Carpels	This plant does not produce fruit and it is propagated by grafting method Carpels: sterile, most abortive with few ripening Ripe carpels: ovoid to ellipsoid Length: 5 mm

**Table 2:** Antimicrobial activities screening from different part of *Michelia x alba* plant.

Plant Part	Types of Extract	Types of Antimicrobial Assay and Pathogens Test	References
**Antibacterial and antifungal**			
Flower	Essential oil	Well diffusion—*A. flavus*	[11,39]
Leaves and stems	Essential oil	Disc diffusion—*S. aureus* ATCC 13709; *E. coli* ATCC 25922; *Candida albican* ATCC 10231	[32]
Bark	Crude methanol extract	Well diffusion—*Curvularia verruculosa*	[34]
Leaf	Essential oil extract in dichloromethane	Disc diffusion and in vitro assay—*Psedumonas aeuroginosa* and *C. albican*; disc diffusion and in vitro assay—*F. oxysporium*	[33]
Flower	Extract		
-	Essential oil	In vitro assay: *A. niger*, *A. flavus*, *Penicillium* sp., *Rhizopus* sp., *Fusarium* sp. and *Cladosporium* sp.	[35]
-	Essential oil	Agar plate of spore and mycellium of *A. flavus* WU 1511	[11]
Flower	Essential oil	Disc diffusion: *S. aureus* and *E. coli*	[40]
**Antiparasitics**			
Bark	Caryophyllene oxide, costunolide, dihydrocostunolide, parthenolide, dihydroparthenolide, 11,13-dehydrolanuginolide, santamarine, and dehydrolinalool oxide	*Trypanosoma cruzi*	[37]
-	Individual compound isolated from *M. alba*: (−)-anonaine	*Plasmodium falciparum*	[38]

## Data Availability

Not applicable.

## Supplementary Materials

The following supporting information can be downloaded at: https://www.mdpi.com/article/10.3390/molecules27113450/s1, Supplementary Data S1: The constituents identified from the essential oil (EO) extracted from flower (F), leaves (L), and stem (S) of *Michelia alba* and Supplementary Data S2: Constituents identified from the extracts of different plant part of *M. alba.*
