# Supplementary material for: Phytochemistry, Bioactivities and Traditional Uses of Michelia × alba
Source: Molecules. 2022 May 26;27(11):3450. doi: 10.3390/molecules27113450 (PMC9182571; doi:10.3390/molecules27113450)
Supplement: Supplementary file 1 [file molecules-27-03450-s001.zip › Supplementary Data S1 - Molecule.pdf]

The constituents identified from the essential oil (EO) extracted from flower (F), leaves (L) and stem (S) of *Michelia alba*

Class	**Constituents**	Chemical structure	**F**	**L**	**S**	**Types of EO**	**Reference**
Monoterpene	1. camphene	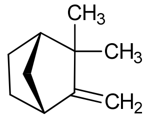	/	/	/	steam distillation	(Bi-yao and Dan, 1982; Huang *et al.*, 2009a; Huang *et al.*, 2009b)
	2. camphor	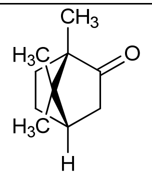	/			Thai China Flavors & Fragrances Co., Ltd	(Songsamoe *et al.*, 2017)
	3. (+)-2-bornanone	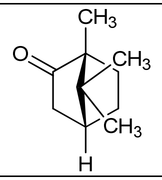		/	/	steam distillation	(Huang *et al.*, 2009a)
	4. borneol	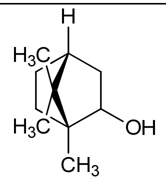	/	/	/	steam distillation	(Huang *et al.*, 2009a; Huang *et al.*, 2009b)
	5. borneol, acetate	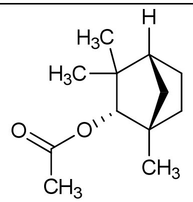		/	/	steam distillation	(Huang *et al.*, 2009a)

	6. 3-carene	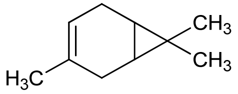	/			steam distillation	(Bi-yao and Dan, 1982)
	7. trans-carveol	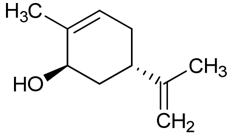	/			steam distillation	(Bi-yao and Dan, 1982)
	8. cis-citral	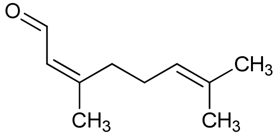		/	/	steam distillation	(Huang *et al.*, 2009a)
	9. trans-citral	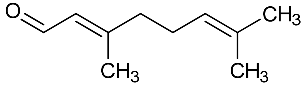		/	/	steam distillation	(Huang *et al.*, 2009a)
	10. eucalyptol / 1,8-cineole	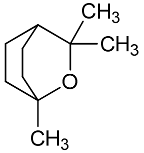	/	/	/	steam distillation	(Huang *et al.*, 2009a) (Bi-yao and Dan, 1982; Huang *et al.*, 2009a; Huang *et al.*, 2009b)
	11. α-fenchene	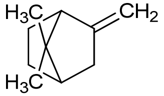	/	/		steam distillation	(Huang *et al.*, 2009b)
	12. (R)-fenchone	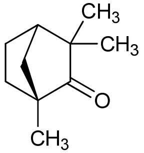	/			Thai China Flavors & Fragrances Co., Ltd	(Songsamoe *et al.*, 2017)

	13. geranyl acetate	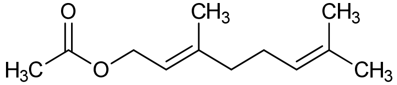		/		steam distillation	(Huang *et al.*, 2009b)
	14. (2Z,6Z)-octa-2,6-dien-1-y acetate	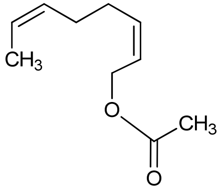	/	/	/	steam distillation	(Huang *et al.*, 2009a; Huang *et al.*, 2009b)
	15. *cis*-geraniol	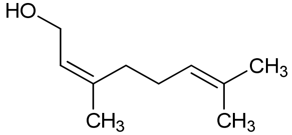	/	/	/	steam distillation	(Huang *et al.*, 2009a; Huang *et al.*, 2009b)
	16. *trans*-geraniol	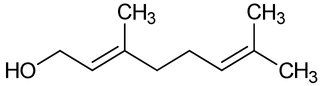	/	/	/	steam distillation	(Huang *et al.*, 2009a; Huang *et al.*, 2009b)
	17. methyl geranate	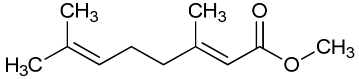		/		steam distillation	(Huang *et al.*, 2009a)
	18. α -limonene diepoxide	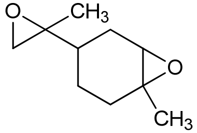			/	steam distillation	(Huang *et al.*, 2009a)
	19. (S)-limonene	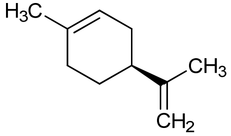	/			Thai China Flavors & Fragrances Co., Ltd	(Songsamoe *et al.*, 2017)
	20. limonene	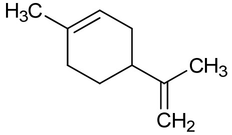	/			steam distillation	(Bi-yao and Dan, 1982)
	21. epoxylinalool	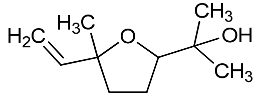	/	/	/	hexane extraction,	(Pensuk *et al.*, 2007; Huang *et al.*,

						steam distillation	2009a; Huang *et al.*, 2009b)
	22. limonene oxide	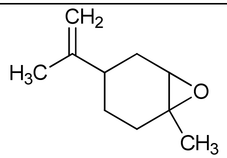	/	/		steam distillation	(Huang *et al.*, 2009b)
	23. cis-linalool oxide	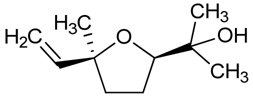	/	/		steam distillation	(Bi-yao and Dan, 1982; Pensuk *et al.*, 2007; Huang *et al.*, 2009a; Huang *et al.*, 2009b)
	24. trans-linalool oxide	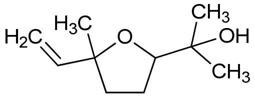	/	/	/	steam distillation	(Bi-yao and Dan, 1982; Pensuk *et al.*, 2007; Huang *et al.*, 2009a; Huang *et al.*, 2009b)
	25. linalool	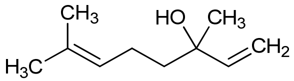	/	/	/	steam distillation	(Bi-yao and Dan, 1982; Pensuk *et al.*, 2007; Huang *et al.*, 2009a; Huang *et al.*, 2009b; Songsamoe *et al.*, 2017)

	26. 8-hydroxy linalool	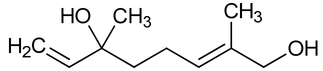			/	steam distillation	(Huang *et al.*, 2009a)
	27. cis-p-2,8-menthadien-1-ol	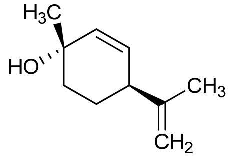			/	steam distillation	(Huang *et al.*, 2009a)
	28. α-myrcene	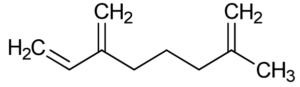	/	/			(Huang *et al.*, 2009b; Songsamoe *et al.*, 2017)
	29. β-myrcene	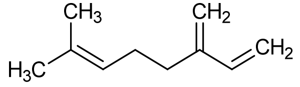	/	/	/	steam distillation	(Bi-yao and Dan, 1982; Huang *et al.*, 2009a)
	30. nopol	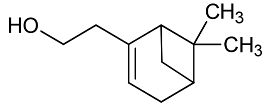		/		steam distillation	(Huang *et al.*, 2009b)
	31. cis-ocimene / (Z)- ocimene	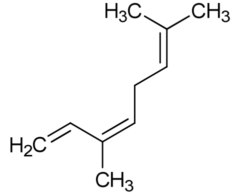	/			steam distillation, hexane extraction	(Pensuk *et al.*, 2007)
				/	/	steam distillation	(Huang *et al.*, 2009a)
	32. trans-ocimene / (E)-ocimene	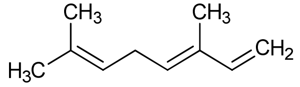	/			steam distillation, hexane extraction	(Pensuk *et al.*, 2007)

			/	/	/	steam distillation	(Huang *et al.*, 2009a; Huang *et al.*, 2009b)
	33. phellandrene	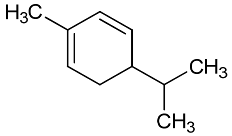	/			steam distillation	(Bi-yao and Dan, 1982)
	34. α-pinene	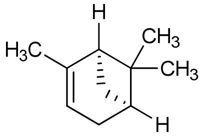	/	/	/	steam distillation	(Huang *et al.*, 2009a; Huang *et al.*, 2009b)
	35. β-pinene	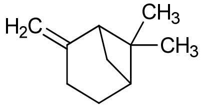	/	/	/	steam distillation	(Bi-yao and Dan, 1982; Huang *et al.*, 2009a)
	36. pinen-3-one	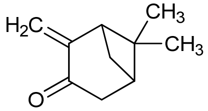			/	steam distillation	(Huang *et al.*, 2009a)
	37. (+)-sabinene	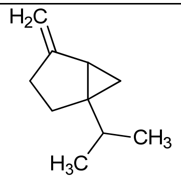	/	/	/	steam distillation	(Huang *et al.*, 2009a; Huang *et al.*, 2009b)
	38. γ-terpinene	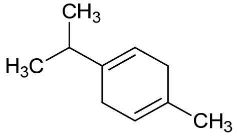	/			steam distillation	(Huang *et al.*, 2009b)

	39. α-terpineol	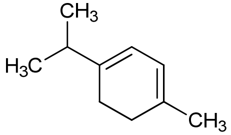		/		steam distillation	(Huang *et al.*, 2009b)
	40. cis-β-terpineol	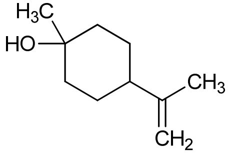	/	/		steam distillation	(Huang *et al.*, 2009b)
	41. 4-terpineol	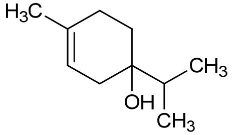	/	/	/	steam distillation	(Huang *et al.*, 2009a; Huang *et al.*, 2009b)
	42. 2,3,3- trimethyl-2-norbornanol	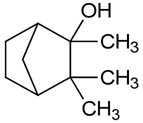	/	/		steam distillation	(Huang *et al.*, 2009b)
sesquiterpene	43. α-cadinol	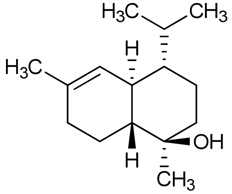		/	/	steam distillation	(Huang *et al.*, 2009a)
	44. T-cadinol	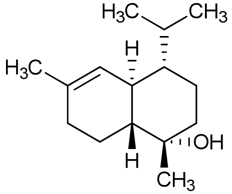			/	steam distillation	(Huang *et al.*, 2009a)
	45. aromadendrene	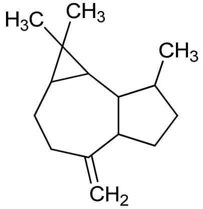		/	/	steam distillation	(Huang *et al.*, 2009a; Huang *et al.*, 2009b)

	46. (-)-isoaromadendrene	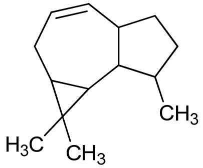	/			steam distillation	(Huang *et al.*, 2009b)
	47. isoaromadendrene epoxide	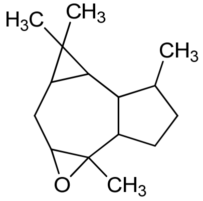	/	/	/	steam distillation	(Huang *et al.*, 2009a; Huang *et al.*, 2009b)
	48. decahydro-1,1,7-trimethyl 4-methylene-1 H-cycloprop[e]azulene	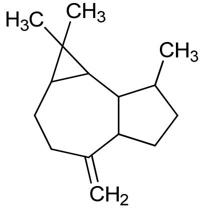	/			steam distillation	(Huang *et al.*, 2009b)
	49. aristolene	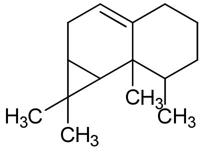	/			steam distillation	(Bi-yao and Dan, 1982)
	50. β-bisabolene	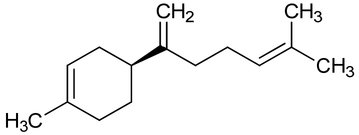	/		/	steam distillation	(Bi-yao and Dan, 1982; Huang *et al.*, 2009a)
	51. trans-*Z*-α -bisabolene epoxide	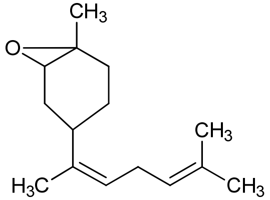		/	/	steam distillation	(Huang *et al.*, 2009a)

	52. α-cadinene	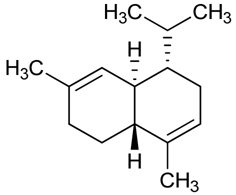	/	/		steam distillation	(Huang *et al.*, 2009b)
	53. δ-cadinene	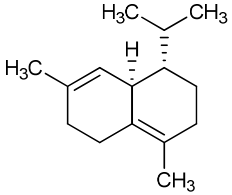	/			steam distillation	(Bi-yao and Dan, 1982)
	54. (+)-δ-cadinene	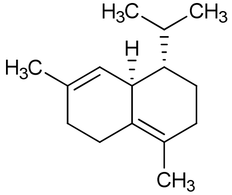		/	/	steam distillation	(Huang *et al.*, 2009a)
	55. 1α-cadin-4-en-10-ol	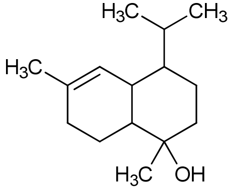		/		steam distillation	(Huang *et al.*, 2009b)
	56. trans-caryophyllene	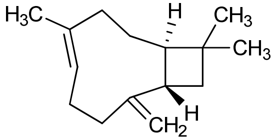	/			steam distillation	(Pensuk *et al.*, 2007)

	57. cis-caryophyllene	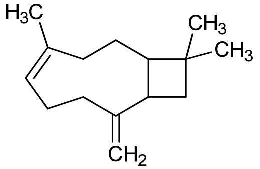	/			steam distillation	(Bi-yao and Dan, 1982)
	58. caryophyllene oxide	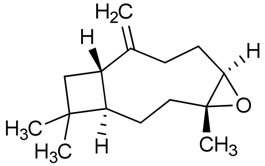	/	/	/	steam distillation	(Huang *et al.*, 2009a; Huang *et al.*, 2009b)
	59. α-chamigrene	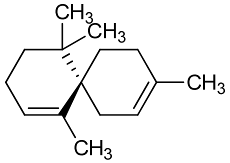	/	/		steam distillation	(Huang *et al.*, 2009b)
	60. β-chamigrene	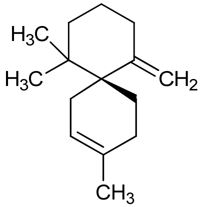		/	/		(Huang *et al.*, 2009a)
	61. copaene	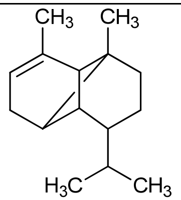		/	/	steam distillation	(Huang *et al.*, 2009a; Huang *et al.*, 2009b)
	62. α-copaen-11-ol	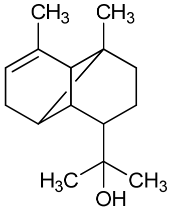	/	/	/	steam distillation	(Huang *et al.*, 2009a; Huang *et al.*, 2009b)

	63. α-cubebene	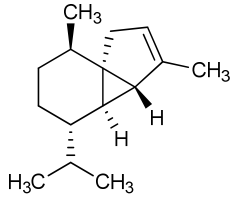	/	/	/	steam distillation	(Bi-yao and Dan, 1982; Huang *et al.*, 2009a; Huang *et al.*, 2009b)
	64. β-cubebene	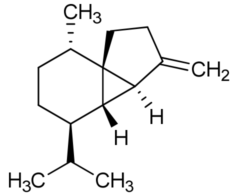	/	/	/	steam distillation	(Huang *et al.*, 2009a) (Bi-yao and Dan, 1982; Huang *et al.*, 2009a)
	65. cubenol	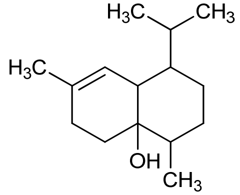	/	/	/	steam distillation	(Bi-yao and Dan, 1982; Huang *et al.*, 2009a; Huang *et al.*, 2009b)
	66. eudesm-7(11)-en-4-ol	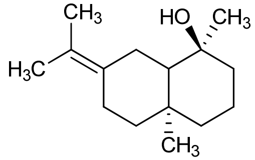	/	/		steam distillation	(Huang *et al.*, 2009a; Huang *et al.*, 2009b)
	67. β-elemene	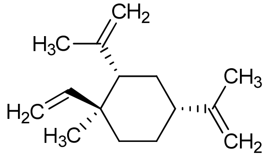	/			steam distillation	(Huang *et al.*, 2009b)
	68. δ-elemene	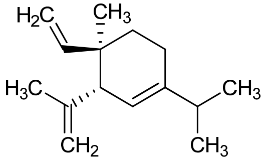	/			steam distillation	(Huang *et al.*, 2009b)

	69. epiglobulol	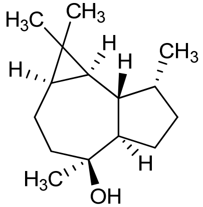	/	/	/	steam distillation	(Huang *et al.*, 2009a; Huang *et al.*, 2009b)
	70. eremophila-1(10),11 -diene	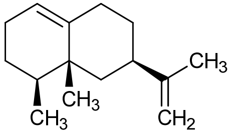		/		steam distillation	(Huang *et al.*, 2009b)
	71. α-farnesene	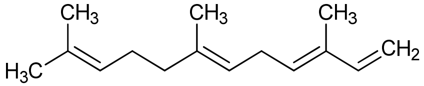	/			steam distillation	(Huang *et al.*, 2009b)
	72. *E*-farnesene epoxide	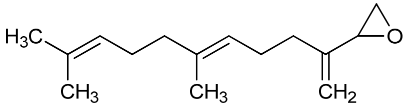		/		steam distillation	(Huang *et al.*, 2009a; Huang *et al.*, 2009b)
	73. (2*Z*, 6*E*)-farnesol	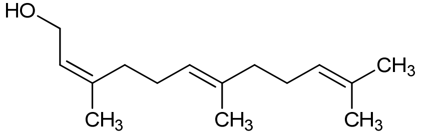	/	/		steam distillation	(Huang *et al.*, 2009b)
	74. (2*E*, 6*E*)-farnesol	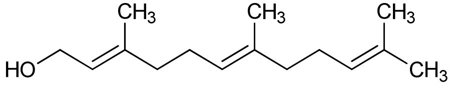		/		steam distillation	(Huang *et al.*, 2009b)
	75. cis farnesol	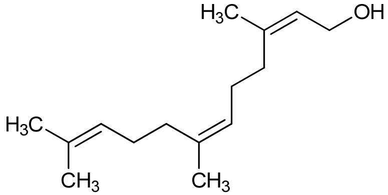		/		steam distillation	(Huang *et al.*, 2009a)
	76. trans-farnesol	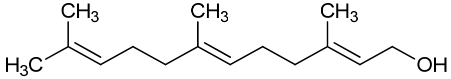		/	/	steam distillation	(Huang *et al.*, 2009a)

	77. germacrene D	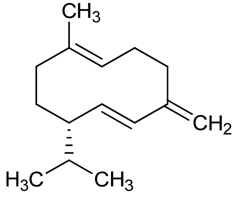	/	/	/	steam distillation, enfleurage method	(Pensuk *et al.*, 2007; Huang *et al.*, 2009a; Huang *et al.*, 2009b)
	78. 8-isopropenyl-1,5-dimethyl-cyclodeca-1,5-diene	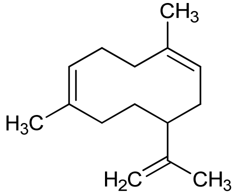	/	/		steam distillation	(Huang *et al.*, 2009a; Huang *et al.*, 2009b)
	79. α-humulene	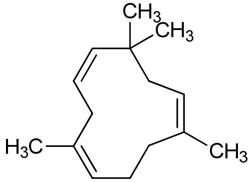	/	/	/	steam distillation	(Huang *et al.*, 2009a; Huang *et al.*, 2009b)
	80. isoledene	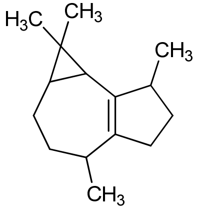	/	/	/	steam distillation	(Huang *et al.*, 2009a; Huang *et al.*, 2009b)
	81. ledene oxide-(II)	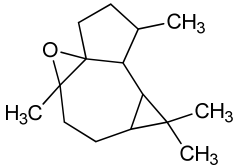			/	steam distillation	(Huang *et al.*, 2009a)
	82. ledol	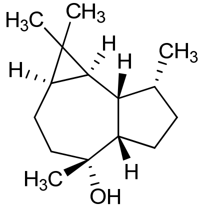		/	/	steam distillation	(Huang *et al.*, 2009a; Huang *et al.*, 2009b)

	83. longipinocarvone	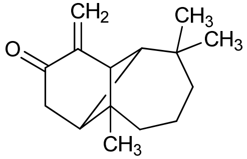	/	/		steam distillation	(Huang *et al.*, 2009b)
	84. trans-longipinocarveol	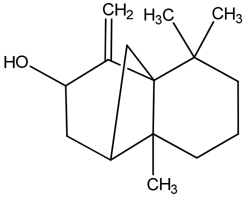		/	/	steam distillation	(Huang *et al.*, 2009a)
	85. β-muurolene	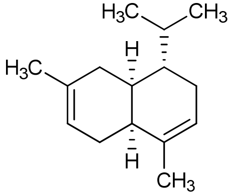			/	steam distillation	(Huang *et al.*, 2009a)
	86. γ-muurolene	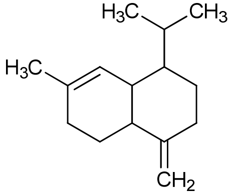			/	steam distillation	(Huang *et al.*, 2009a)
	87. T-muurolol	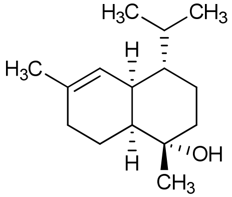			/	steam distillation	(Huang *et al.*, 2009a)
	88. nerolidol	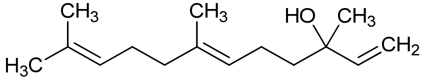	/	/	/	steam distillation	(Huang *et al.*, 2009a; Huang *et al.*, 2009b)

	89. (+)-sativene	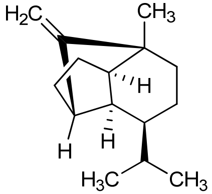	/	/		steam distillation	(Huang *et al.*, 2009b)
	90. sativene	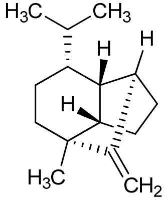			/	steam distillation	(Huang *et al.*, 2009b)
	91. selina-6-en-4-ol	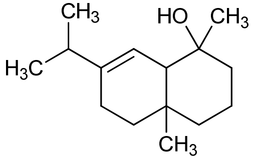	/	/		steam distillation	(Huang *et al.*, 2009b)
	92. α-selinene	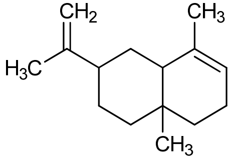	/	/	/	steam distillation	(Huang *et al.*, 2009a; Huang *et al.*, 2009b)
	93. β-selinene	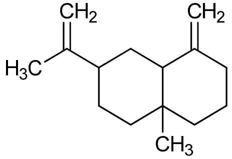	/			steam distillation	(Bi-yao and Dan, 1982)
	94. eudesma-4(14), 11-diene	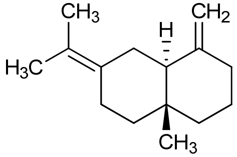	/	/	/	steam distillation	(Huang *et al.*, 2009a; Huang *et al.*, 2009b)

	95. spathulenol	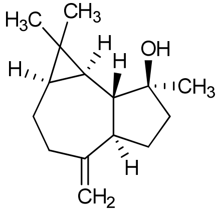	/	/		steam distillation	(Huang *et al.*, 2009b)
	96. (-)-spathulenol	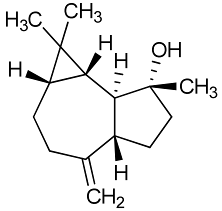		/	/	steam distillation	(Huang *et al.*, 2009a)
	97. (+)-spathulenol	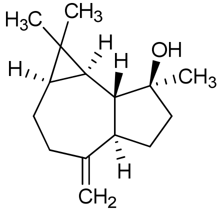		/	/	steam distillation	(Huang *et al.*, 2009a)
	98. (5*E*,9*E*)-12-methyl-1,5,9,11-tridecatetraene	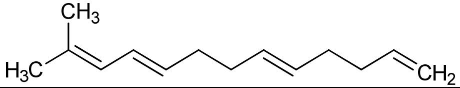	/	/		steam distillation	(Huang *et al.*, 2009b)
	99. viridiflorol	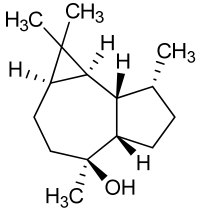	/	/		steam distillation	(Huang *et al.*, 2009b)
	100. widdrol	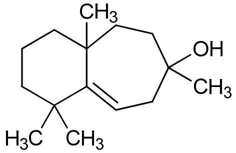			/	steam distillation	(Huang *et al.*, 2009a)

	101. α-ylangene	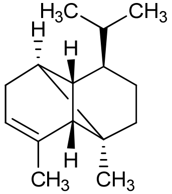	/			steam distillation	(Bi-yao and Dan, 1982)
Diterpene	102. phytol	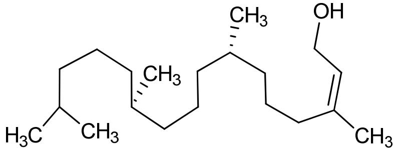		/		steam distillation	(Huang *et al.*, 2009b)
Macro Diterpene	103. β-4,8,13-duvatriene-1,3-diol	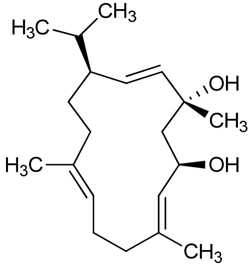			/	steam distillation	(Huang *et al.*, 2009a)
fatty acid	104. benzenepropanoic acid, methyl ester	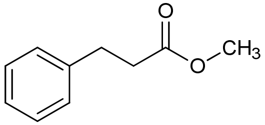	/			steam distillation	(Huang *et al.*, 2009b)
	105. 2-hydroxy-benzoic acid methyl ester	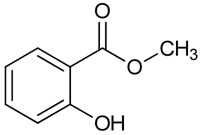		/	/	steam distillation	(Huang *et al.*, 2009a; Huang *et al.*, 2009b)
	106. 2-methyl butanoic acid	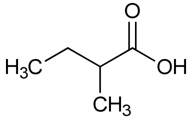	/			enfleurage method, hexane extraction	(Pensuk *et al.*, 2007)

	107. 11,14,17-eicosatrienoic acid, methyl ester	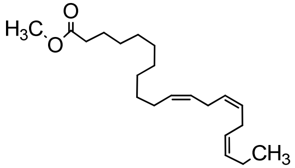		/			
	108. ethyl-2-methyl butyrate	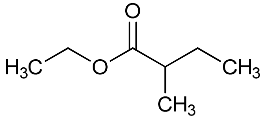	/			steam distillation, enfleurage method,	(Pensuk *et al.*, 2007)
	109. methyl 2-methylbutyrate	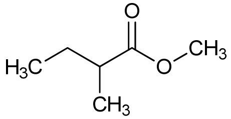	/			steam distillation, enfleurage method, Water distillation	(Bi-yao and Dan, 1982) (Pensuk *et al.*, 2007; Punjee *et al.*, 2009)
	110. 2-methylbutyl-2-methylbutyrate	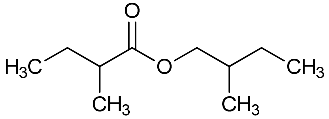	/			enfleurage method	(Pensuk *et al.*, 2007)
	111. phenylethyl-2-methyl butyrate	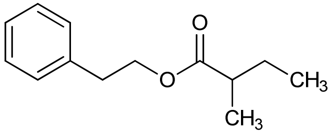	/			enfleurage method	(Pensuk *et al.*, 2007)
	112. palmitic acid / hexadecanoic acid / *n*-hexadecanoic acid	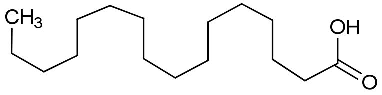	/			enfleurage method, steam distillation	(Pensuk *et al.*, 2007; Huang *et al.*, 2009b)
				/		steam distillation	(Huang *et al.*, 2009b)
	113. nonadecanoic acid	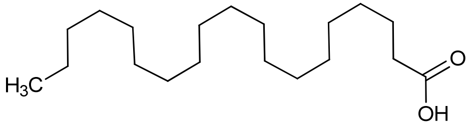		/	/	steam distillation	(Huang *et al.*, 2009a)

	114. linolenic acid, methyl este	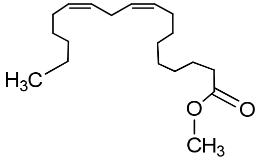	/	/		steam distillation	(Huang *et al.*, 2009b)
	115. 9,12-octadecadienoic acid methyl ester	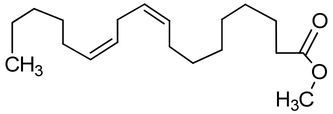	/	/		steam distillation	(Huang *et al.*, 2009b)
Fatty acid methyl ester	116. 13-docosenoic acid methyl ester	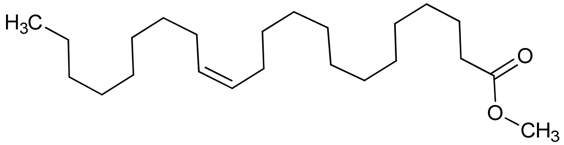			/	steam distillation	(Huang *et al.*, 2009a)
Alcohol	117. phenylethyl alcohol	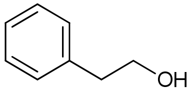	/			steam distillation	(Pensuk *et al.*, 2007)
	118. 2-octenol	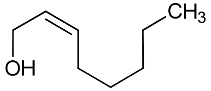		/		steam distillation	(Huang *et al.*, 2009b)
	119. (9*E*,12*E*,15*E*)-9,12,15-octadecatrien-1-ol	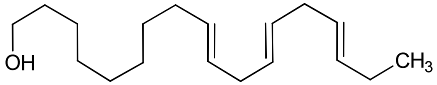	/	/	/	steam distillation	(Huang *et al.*, 2009a; Huang *et al.*, 2009b)
	120. 9,12,15-octadecatrien -1-ol	Same as #95	/	/		steam distillation	(Huang *et al.*, 2009b)
allyl	121. *p*-propenylanisole	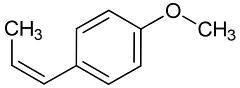		/	/	steam distillation	(Huang *et al.*, 2009a; Huang *et al.*, 2009b)
allylbenzene	122. eugenol	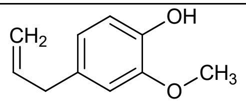	/		/	steam distillation, MeOH extract	(Bi-yao and Dan, 1982; Pensuk *et al.*, 2007; Lo *et al.*, 2010)

alkyl	123. 2-undecanone	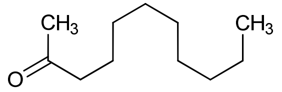	/	/	/	steam distillation	(Huang *et al.*, 2009a; Huang *et al.*, 2009b)
	124. 6,10-dimethyl-2-undecanone	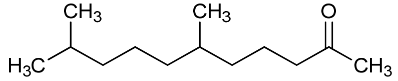			/	steam distillation	(Huang *et al.*, 2009a)
alkane	125. eicosane	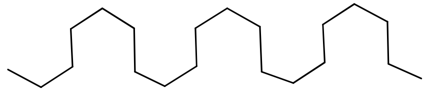		/	/	steam distillation	(Huang *et al.*, 2009a)
phenylpropanoid	126. α-asarone	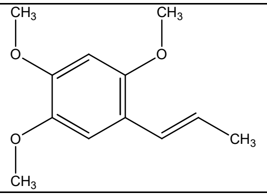			/	steam distillation	(Huang *et al.*, 2009a)
	127. methyl isoeugenol / Isoeugenyl methyl ether	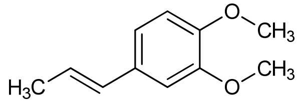	/			steam distillation	(Bi-yao and Dan, 1982)
			/	/	/	steam distillation	(Huang *et al.*, 2009a; Huang *et al.*, 2009b)
	128. methyl eugenol / eugenol methyl ether	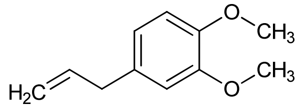	/	/		steam distillation	Bi-yao and Dan, 1982) (Huang *et al.*, 2009a; Huang *et al.*, 2009b)
			/			hexane extraction	(Pensuk *et al.*, 2007)
polycyclic heteroarene	129. indole (1H-indole)	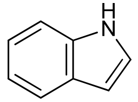	/	/		enfleurage method, steam distillation	(Pensuk *et al.*, 2007; Huang *et al.*, 2009b)

ester	130. methyl benzoate	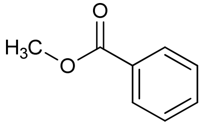	/			hexane extraction	(Pensuk *et al.*, 2007)
	131. phenyl-2-methyl benzoate	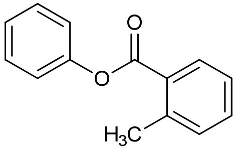	/			hexane extraction	(Pensuk *et al.*, 2007)
	132. acetic acid, 2-phenyl ethyl ester	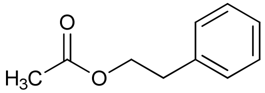	/			steam distillation	(Huang *et al.*, 2009b)
	133. propanoic acid, 2-phenylethyl ester	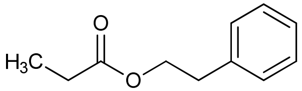	/			steam distillation	(Huang *et al.*, 2009b)
bicyclic aromatic organic compound	134. phthalan /1,3-dihydroisobenzofuran	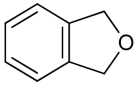	/	/		steam distillation	(Huang *et al.*, 2009b)
Aldehyde	135. butanal	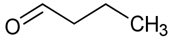		/		steam distillation	(Huang *et al.*, 2009a)
	136. benzeneacetaldehyde	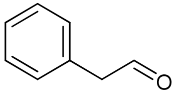			/	steam distillation	(Huang *et al.*, 2009a)
	137. octanal	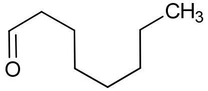		/		steam distillation	(Huang *et al.*, 2009b)
Alkyl-Aldehyde	138. pentanal	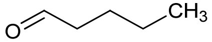		/		steam distillation	(Huang *et al.*, 2009a)
Hydrocarbon	139. estragole	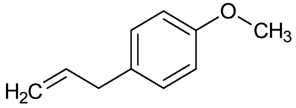	/	/	/	steam distillation	(Huang *et al.*, 2009a; Huang *et al.*, 2009b)
	140. *n*-heptacosane	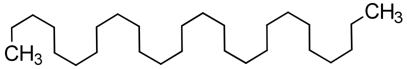	/	/			

	141. *n*-octacosane	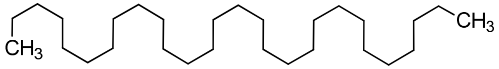	/	/		steam distillation	(Huang *et al.*, 2009b)
	142. 1,5,5-trimethyl-6-methylene-1-cyclo hexane	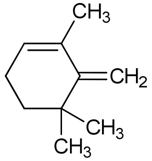	/			steam distillation	(Huang *et al.*, 2009b)
	143. *n*-pentadecane	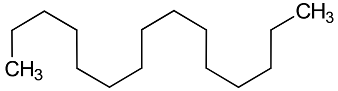	/	/		steam distillation	(Huang *et al.*, 2009b)
	144. 2,4-diisopropenyl-1-methyl-1-vinylcyclohexane	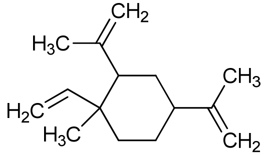	/	/		steam distillation	(Huang *et al.*, 2009b)
	145. (2α, 3β, 5β)-1,1,2-trimethyl-3,5-bis(1-methylethenyl)-cyclo hexane	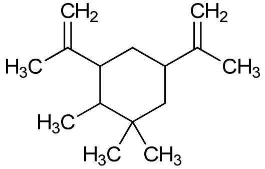	/			steam distillation	(Huang *et al.*, 2009b)
fatty alcohols	146. (+,-)-nuciferol	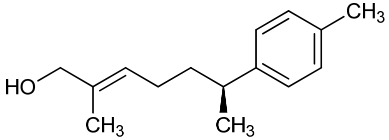	/			steam distillation	(Huang *et al.*, 2009b)
Bezene	147. 5-(2-propenyl)-1, 3-benzodioxole	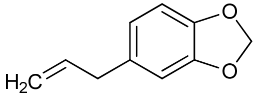	/	/		steam distillation	(Huang *et al.*, 2009b)
Phenols	148. 2-methoxy-4-vinyl phenol	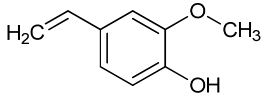	/			steam distillation	(Huang *et al.*, 2009b)
Ketone	149. 1,2,3,5,6,7-hexahydro-inden-4-one	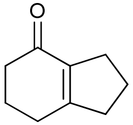		/		steam distillation	(Huang *et al.*, 2009b)

Monoterpene	150. 2-(acetylmethyl)-(+)-3-carene	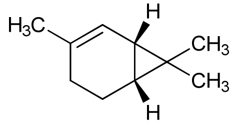	/			steam distillation	(Huang *et al.*, 2009b)
Monoterpene	151. 10-(acetylmethyl)-(+)-3-carene	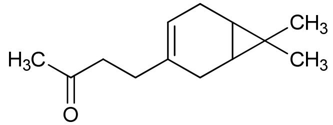		/	/	steam distillation	(Huang *et al.*, 2009a)
Monoterpene	152. ocimenol	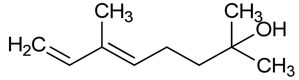		/	/	steam distillation	(Huang *et al.*, 2009a)
Ester	153. 3-methyl-2-butenoic acid,-phenylethyl ester	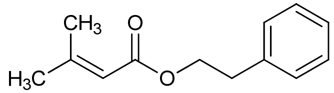		/	/	steam distillation	(Huang *et al.*, 2009a)
Ketone	154. 4-Camphenylbutan -2-one	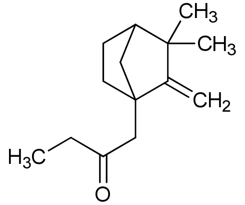			/	steam distillation	(Huang *et al.*, 2009a)
Alcohol	155. Z,Z-8,10-Hexadecadien -1-ol	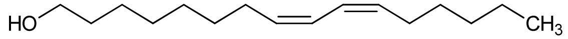			/	steam distillation	(Huang *et al.*, 2009a)
Alkane	156. 6-methyloctadecane	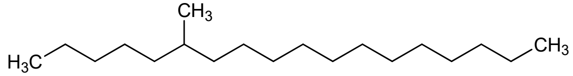			/	steam distillation	(Huang *et al.*, 2009a)
Diester	157. butyl octyl phthalate	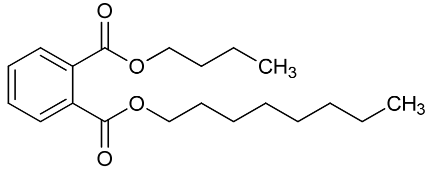		/	/	steam distillation	(Huang *et al.*, 2009a)
