# Supplementary material for: Phytochemistry, Bioactivities and Traditional Uses of Michelia × alba
Source: Molecules. 2022 May 26;27(11):3450. doi: 10.3390/molecules27113450 (PMC9182571; doi:10.3390/molecules27113450)
Supplement: Supplementary file 1 [file molecules-27-03450-s001.zip › Supplementary Data S2 - Molecule.pdf]

Supplementary Data S2: Constituents identified from the extracts of different plant part of *M. alba*

| | Constituents | Chemical structure | F | L | S |
|---|---|---|---|---|---|
| aporphines | 1. (-)-anonaine | 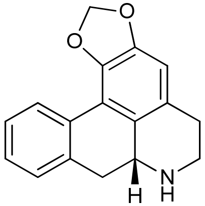 | / | / | / |
| | 2. (-)-norushinsunine | 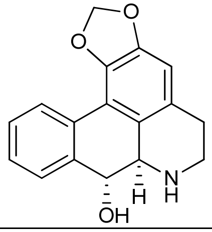 | / | / | / |
| | 3. (-)-ushinsunine | 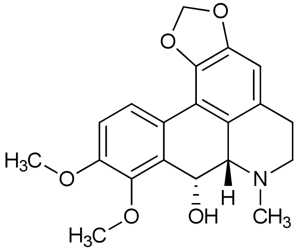 | / | / | / |
| | 4. (-)-N-acetylanonaine | 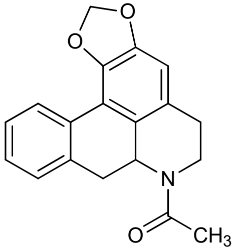 | / | / | |

| | | | | | |
|---|---|---|---|---|---|
| | 5. (–)-*N*-formylanonaine | 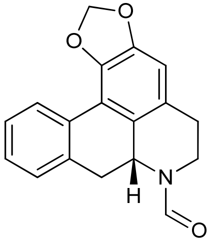 | | | / |
| | 6. (–)-romerine | 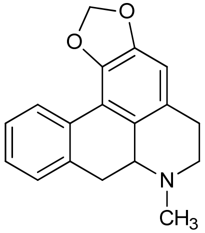 | | | / |
| | 7. (–)-asimilobine | 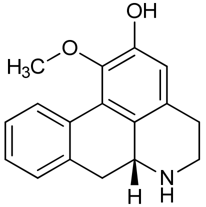 | | | / |
| oxoaporphines | 8. Liriodenine | 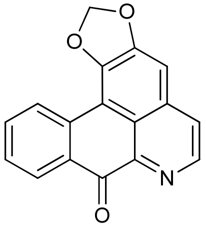 | / | / | / |
| | 9. Oxoxylopine | 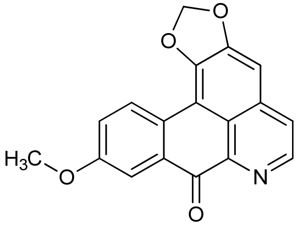 | | / | / |

| | | | | | |
|---|---|---|---|---|---|
| | 10. atherospermidine | 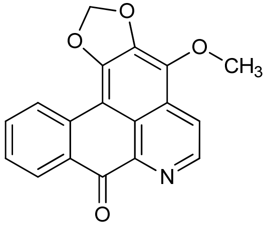 | / | | |
| 4,5-dioxoaporphine | 11. michelaine | 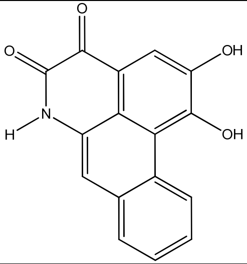 | / | | |
| sesquiterpene lactones | 12. michelenolide | 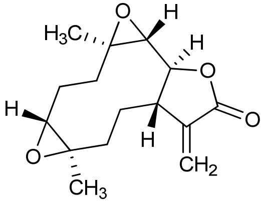 | | / | |
| | 13. Costunolide | 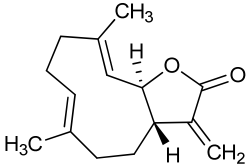 | | / | |
| | 14. 11,13-dehydrolanuginolide | 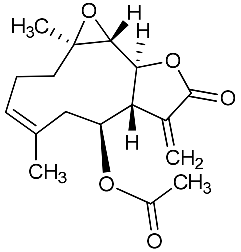 | | / | |

| | | | | | |
|---|---|---|---|---|---|
| amide | 15. N-trans-feruloyltyramine | 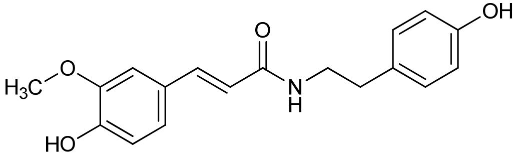 | / | / | / |
| lignan | 16. (+)-syringaresinol | 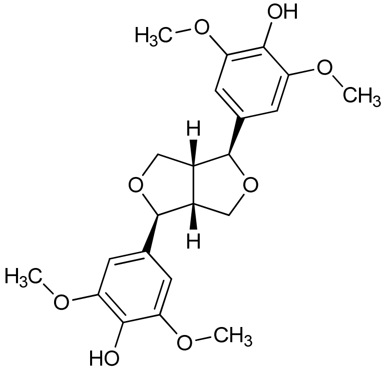 | / | / | / |
| benzenoids | 17. 4-hydroxybenzaldehyde | 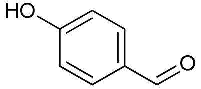 | | / | / |
| | 18. 4-hydroxybenzoic acid | 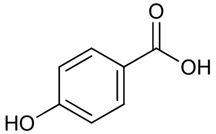 | | / | |
| | 19. methylparaben | 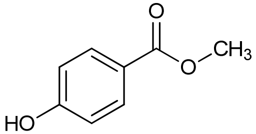 | | / | |
| | 20. *p*-anisaldehyde | 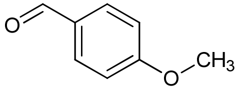 | | | / |
| | 21. veratraldehyde | 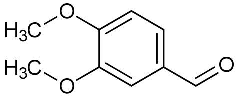 | | | / |

| | | | | | |
|---|---|---|---|---|---|
| | 22. 3,4,5-trimethoxybenzoic acid | 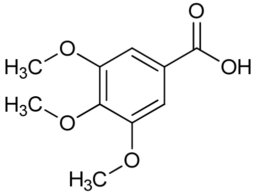 | | | / |
| | 23. 3,4-dimethoxybenzoic acid | 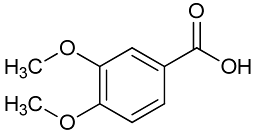 | | | / |
| | 24. eugenol | 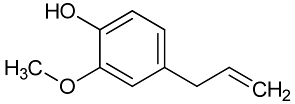 | | | / |
| | 25. methyl isoeugenol | 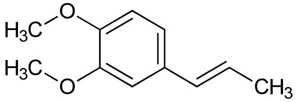 | | | / |
| | 26. *p*-hydroxybenzaldehyde | 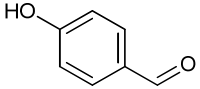 | / | | |
| | 27. vanillin | 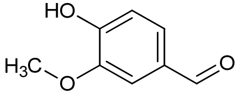 | / | | |
| | 28. vanillic acid | 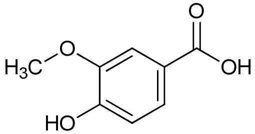 | / | | |
| | 29. syringic acid | 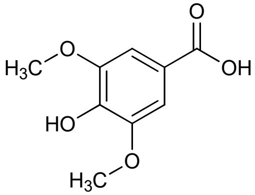 | / | | |
| | 30. coniferyl aldehyde | 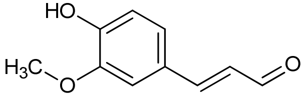 | / | | |

| | | | | | |
|---|---|---|---|---|---|
| | 31. syringin | 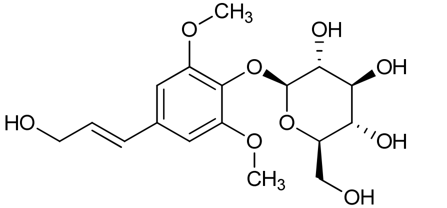 | / | | |
| steroids | 32. b-sitosterol | 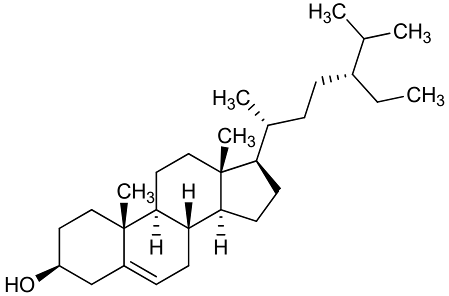 | / | / | / |
| | 33. Stigmasterol | 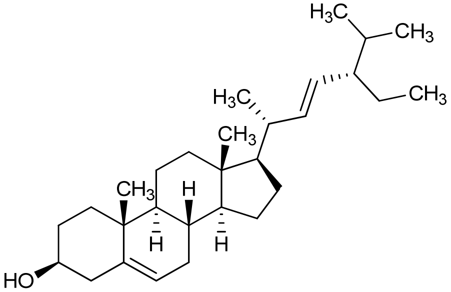 | | / | / |
| | 34. stigmasta-4,22-dien-3-one | 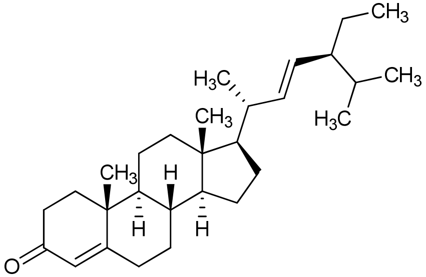 | / | | |

| | | | | | |
|---|---|---|---|---|---|
| aliphatic compounds | 35. palmitic acid | 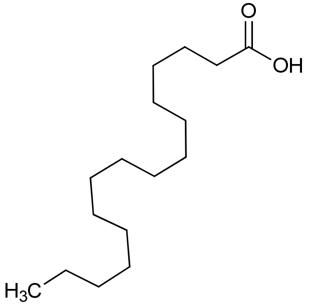 | | / | |
| | 36. stearic acid | 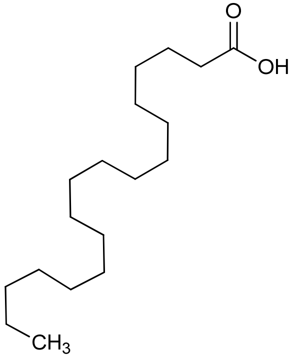 | | / | |
| | 37. linoleic acid | 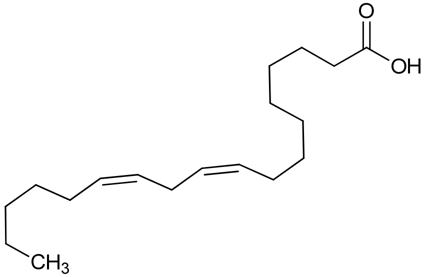 | | / | |

| | | | | | |
|---|---|---|---|---|---|
| chlorophylls | 38. pheophorbide a | 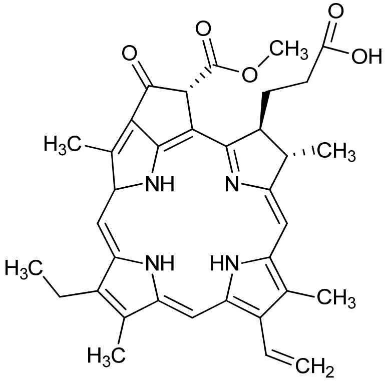 | | / | |
| | 39. aristophyll-C | 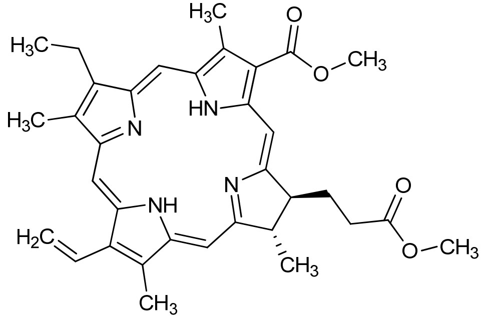 | | / | |
| Polyprenols | 40. ficaprenol | 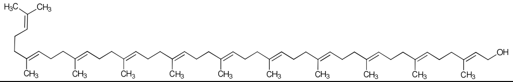 | | | / |
| coumarin | 41. scopoletin | 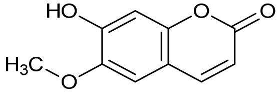 | / | | |
| quinol | 42. 4-acetonyl-3,5-dimethoxy-*p*-quinol | 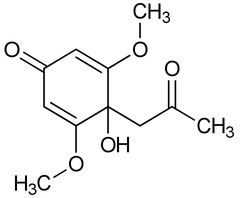 | / | | |
